# Powder diffraction data beyond the pattern: a practical review

**DOI:** 10.1107/S1600576725004728

**Published:** 2025-07-22

**Authors:** Nicola Casati, Elena Boldyreva

**Affiliations:** a Paul Scherrer Institute 111 Forschungstrasse 5232Villigen Switzerland; b Boreskov Institute of Catalysis RAS, Lavrentiev Ave. 5, Novosibirsk 630090, Russian Federation; c Novosibirsk State University Pirogova 2 Novosibirsk630090 Russian Federation; HPSTAR and Harbin Institute of Technology, People’s Republic of China

**Keywords:** powder diffraction, FAIR data, raw data, 2D to 1D conversion, metadata, particle statistics, images, materials, minerals, high-pressure data, *in situ* mechanochemical studies

## Abstract

We share personal experience in the fields of materials science and high-pressure research, discussing which parameters, in addition to positions of peak maxima and intensities, may be important to control and to document in order to make deposited powder diffraction data reusable, reproducible and replicable. We discuss, in particular, which data can be considered as ‘raw’ and some challenges of revisiting deposited powder diffraction data. We consider procedures such as identifying (‘fingerprinting’) a known phase in a sample, solving a bulk crystal structure from powder data, and analyzing the size of coherently scattering domains, lattice strain, the type of defects or preferred orientation of crystallites. The specific case of characterizing a multi-phase multi-grain sample following *in situ* structural changes during mechanical treatment in a mill or on hydrostatic compression is also examined. We give examples of when revisiting old data adds a new knowledge and comment on the challenges of using deposited data for machine learning.

## Abstract 2

We share personal experience in the fields of materials science and high-pressure research, discussing which parameters are important to control and document in order to make deposited powder diffraction data reusable, reproducible and replicable.

## 1. Introduction

One of the modern trends in various fields of science is to ensure that data obtained as a result of research activities not only are correct and reproducible but also are deposited reliably and are accessible long after the data have been collected, to the original authors and to other members of the community. This approach is represented by the acronym FAIR data, *i.e.* data which meet principles of findability, accessibility, interoperability and reusability (Wilkinson *et al.*, 2016 ▸; Jacobsen *et al.*, 2020 ▸). The necessity to deposit raw data^1^ has been discussed for some time in relation to different research fields. Initially, sharing of raw data was conceived mainly to help replicate an experiment and to improve data analysis. In fact, when speaking of preserving raw data for the sake of testing the reproducibility people often mean the importance of these data for replicability. ‘Reproducibility’ refers to instances in which the original researcher’s data and computer codes are used to regenerate the results, while ‘replicability’ refers to instances in which a researcher collects new data to arrive at the same scientific findings as a previous study [see *e.g.* National Academies of Sciences, Engineering, and Medicine (2019 ▸)]. Preserving old raw data and revisiting them later can be useful, in order to check if they have been processed without errors and in the best possible way. Sometimes, examination of the data by another researcher, or by the same researcher years later, reveals details that skipped attention earlier and, as a result, enables them to suggest a new interpretation of the results of the old experiments. Reprocessing old data may be easier than reproducing the experiment itself, if the sample is difficult to reproduce; some natural samples may be unique and as such not reproducible at all. The arrival of artificial intelligence and machine-learning tools has made sharing scientific data even more important, as new correlations and information – either overlooked, unexpected or simply outside of the scope of the original research teams – could emerge when interrogating many related and shared datasets (Warren, 2018 ▸). A large amount of powder diffraction data obtained years ago could be used for machine learning, provided that the important details of data collection have been documented (Greasley & Hosein, 2023 ▸; Surdu & Győrgy, 2023 ▸; Lee *et al.*, 2022 ▸; Lee *et al.*, 2023 ▸; Suzuki, 2022 ▸; Yanxon *et al.*, 2023 ▸; Marchenko *et al.*, 2025 ▸). This holds also for data that remained unpublished, *e.g.* when the researchers who collected these data could not process them properly because they could not identify the corresponding crystal structures (Parackal *et al.*, 2024 ▸).

The importance of preserving experimental data is well realized by the crystallographic community (Helliwell, 2019 ▸; Helliwell, 2022*a* ▸; Helliwell, 2022*b* ▸; Helliwell, 2024 ▸; Helliwell & Massera, 2022 ▸; Kroon-Batenburg *et al.*, 2024 ▸; Helliwell *et al.*, 2024 ▸). Established crystallographic databases include the Cambridge Structural Database (CSD; Groom *et al.*, 2016 ▸), Protein Data Bank (PDB; https://www.rcsb.org/) and International Centre for Diffraction Data (ICDD; https://www.icdd.com/) Powder Diffraction File databases. These have allowed deposititon of not only the atomic coordinates and unit-cell parameters in a formalized way that enables processing of large amounts of data both manually and in an automatic mode (CSD and PDB) (Allen *et al.*, 2004 ▸; Brown & McMahon, 2002 ▸; Coles *et al.*, 2006 ▸; Gildea *et al.*, 2011 ▸) but also the diffraction data – relative intensities of reflections, their angular positions and *d*-spacings for powder data (ICDD), and structure factors for single-crystal data (CSD, PDB). More and more attention is being paid to preserving primary experimental data, raw data and metadata in macromolecular crystallography (Bernstein *et al.*, 2020 ▸; Helliwell, 2022*a* ▸; Helliwell, 2022*b* ▸; Helliwell *et al.*, 2019 ▸; Kroon-Batenburg, 2023 ▸), high-pressure crystallography (Dziubek, 2020 ▸), small-angle scattering (Giess *et al.*, 2023 ▸) and powder diffraction studies (Aranda, 2018 ▸; Aranda, 2019 ▸), as well as to the interoperability of crystallographic data and databases (Bruno *et al.*, 2017 ▸; Brink *et al.*, 2024 ▸). In 2022 a special section of the journal *IUCrData*, called *Raw Data Letters*, was launched (Kroon-Batenburg *et al.*, 2022 ▸). In 2023 CommDat organized a number of sessions related to raw data archiving and raw data reuse at the IUCr 2023 Congress in Melbourne. These included the workshop *Raw diffraction data reuse: the good, the bad and the challenging*, the microsymposium *Raw diffraction data reuse: warts and all* and the keynote lecture by Andy Götz *Europe’s photon and neutron open science clouds for raw and processed data: aims and achievements to date*. We participated in the workshop and were further invited to share here our thoughts on preservation of raw diffraction data collected from powder samples.

Powder X-ray diffraction analysis is a critical component of materials characterization methodologies. Powder diffraction is used at least as often as single-crystal diffraction is, if not much more often. There are different reasons of this. Sometimes, single crystals cannot be obtained – not only because of poor crystallization but because a phase can exist only as a powder (for example a metastable polymorph formed as a result of a solid-state transformation or a very fast precipitation). A multi-phase sample can be represented only by many particles. Powder diffraction gives data which are representative for all the sample, not for a single particle only. It can be used in order to solve various tasks:

(i) to identify the phases in a sample and, in particular, to analyze the phase composition of multi-component samples (qualitatively and quantitatively),

(ii) to measure the unit-cell parameters and volume and their variations with temperature, pressure, composition of solid solutions, concentration of defects, strain and stress,

(iii) to estimate the size of crystallites and domains,

(iv) to find the preferred orientation of particles,

(v) to estimate the content of the amorphous phase(s) in relation to the crystalline phase(s),

(vi) to solve an unknown crystal structure, if a single crystal cannot be obtained,

(vii) to refine a crystal structure that has been preliminarily roughly solved (*e.g.* on the basis of poor-quality or low-completeness single-crystal data, data coming from electron diffraction, or a predicted model).

One can sometimes hear the opinion that powder diffraction is used mainly in industry and mainly for phase identification, whereas other applications are rare and are interesting exclusively for academic researchers. In this respect, the idea of simply storing a list of peak positions and intensities would be much simpler. Our personal experience with industry in the 21st century, though, is that patterns alongside most metadata are normally archived, at times even straight from the labora­tory information management systems, while even samples in certain cases are stored. This could be for legal reasons or as the economic benefits heavily outweigh the storage costs with­in a company. It is the academic and large-scale facility world, where the data flow can be very large, that still lags behind from this point of view and is the main focus of this work.

The first applications of powder diffraction were aimed at solving unknown structures, not identifying the known phases (Bragg, 1913 ▸; Bragg & Bragg, 1913 ▸). A review dedicated to the first 90 years of powder X-ray diffraction gave convincing evidence of the rapid increase in the number of crystal structures determined (solved and/or refined) from powder diffraction data [see Figs. 3 and 4 of Paszkowicz (2006 ▸)]; since 2006 this trend has become even more pronounced. We have not seen any exact statistics of how widespread other applications are and how the trends of relative frequencies of various applications changed with time. This could be an interesting and instructive statistical study. Our personal experience suggests that identification of known phases in a sample is not the main purpose either of the projects that receive beam-time at large-scale facilities (which are the main providers of data and for which the problem of depositing and reusing raw data is currently discussed) or of academic research. As for industry, though it is in fact interested in qualitative and quantitative phase analysis, there are certain fields (*e.g*. pharmaceuticals, pigments, functional materials, catalysts *etc.*) where structure solution from powder diffraction data and the control of crystallinity, disorder, particle size distribution, strain or stress in the samples become more and more in demand. This is particularly true as industries move towards nanomaterials and nanostructured, modulated and disordered phases. Because of the wealth of information and also its complicated nature, data analysis may take a significant amount of time and may require going back to the original, non-processed data, in order to verify the correctness of previous data processing and analysis and, if necessary, to correct the results of interpreting the data.

This contribution was not viewed by us either as a scientific paper reporting new experimental results or as a classical review. It was planned as a scientific opinion or a scientific commentary, both inviting experts to discussion and sharing some personal experience with those who are just entering the field, or who have experience with collecting data but never thought of which parameters are important to note, document and preserve when depositing the processed diffraction data and/or the diffraction pattern itself.

## 2. Not only peak positions and integral intensities

**Figure 1:**
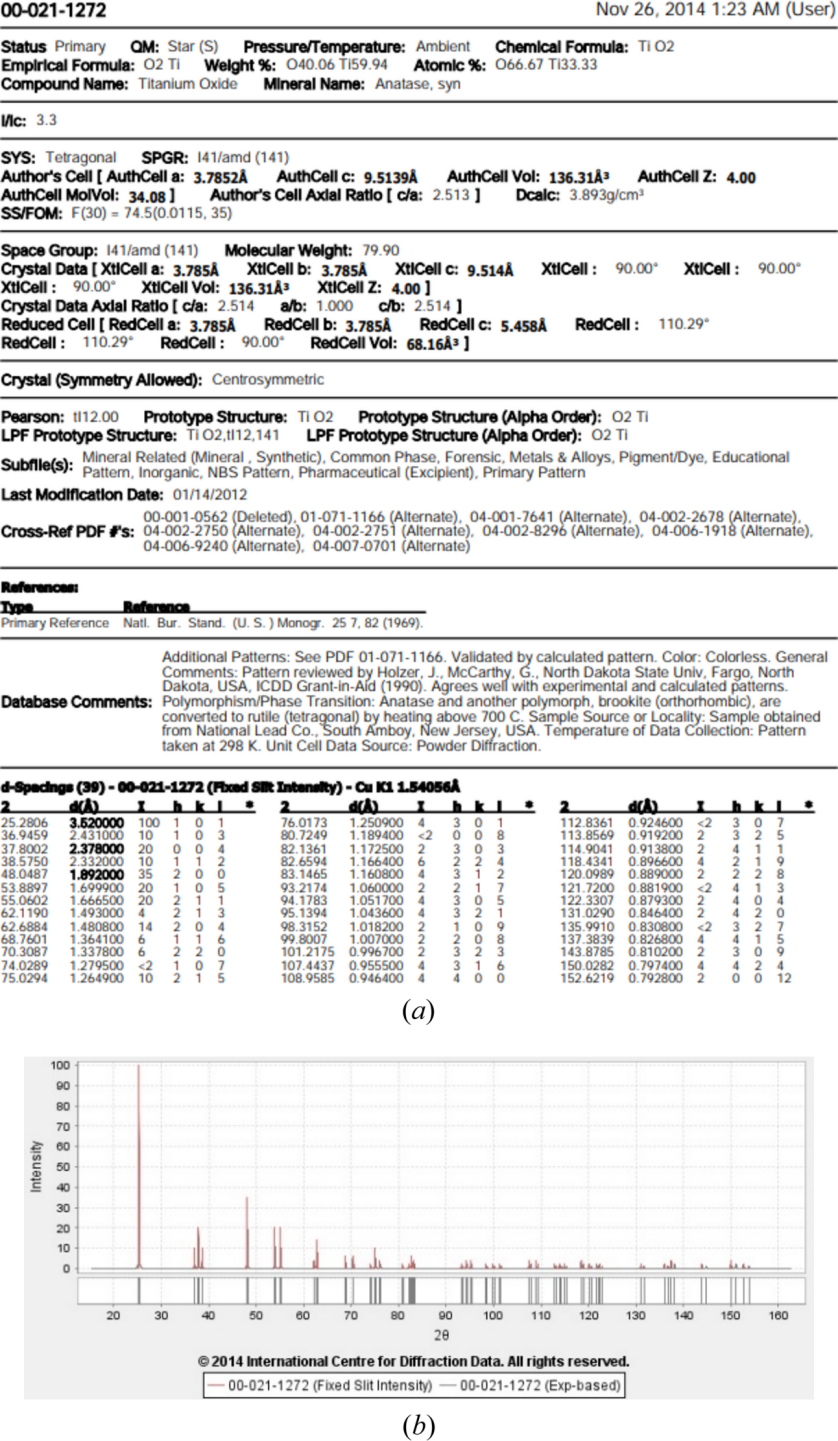
A sample card from the PDF with powder diffraction data for TiO_2_ (anatase). (*a*) Table with 2θ–*d*–*I*–*hkl* data; (*b*) a schematic representation of the powder diffraction pattern.

The early concept of a powder pattern database was limited to depositing the reflections, *d*-spacings and associated relative intensities. For some tasks this information is indeed sufficient even to solve an unknown structure. For example, the first structures solved by the Braggs were relatively simple (diamond, NaCl, ZnS, some other inorganic compounds), and therefore they successfully determined these structures just knowing the interplanar distances exhibited by diffraction patterns and crystalline lattices (Bragg, 1913 ▸; Bragg & Bragg, 1913 ▸). In many cases, it is sufficient to know the positions and intensities of diffraction maxima for identifying known phases (‘fingerprinting’). This is one of the principles on which X-ray diffraction qualitative analysis is based. This was also the concept on which the archiving of powder diffraction data was based when the primary reference of X-ray powder diffraction data, which became known as the Powder Diffraction File (PDF), was created in 1941. This effort was initially supported by the American Society for Testing and Materials (ASTM), and many senior researchers can still remember the ‘ASTM cards’. Over the next two decades, other professional bodies added their support, culminating in 1969 with the establishment of the Joint Committee on Powder Diffraction Standards (JCPDS). The JCPDS was incorporated to continue the mission of maintaining the PDF. In 1978, the name of the organization was changed to the International Centre for Diffraction Data in order to highlight the global commitment of this scientific endeavor [see https://www.icdd.com/assets/history/ICDDHistory-Messick.pdf; https://www.icdd.com/assets/history/100-Years-of-X-ray-Powder-Diffraction-Fawcett.pdf]. Although the information is now stored and transferred electronically, the main principle of storing the powder diffraction data has not changed substantially, and an electronic card from a PDF database looks like that shown in Fig. 1 ▸.

**Figure 2:**
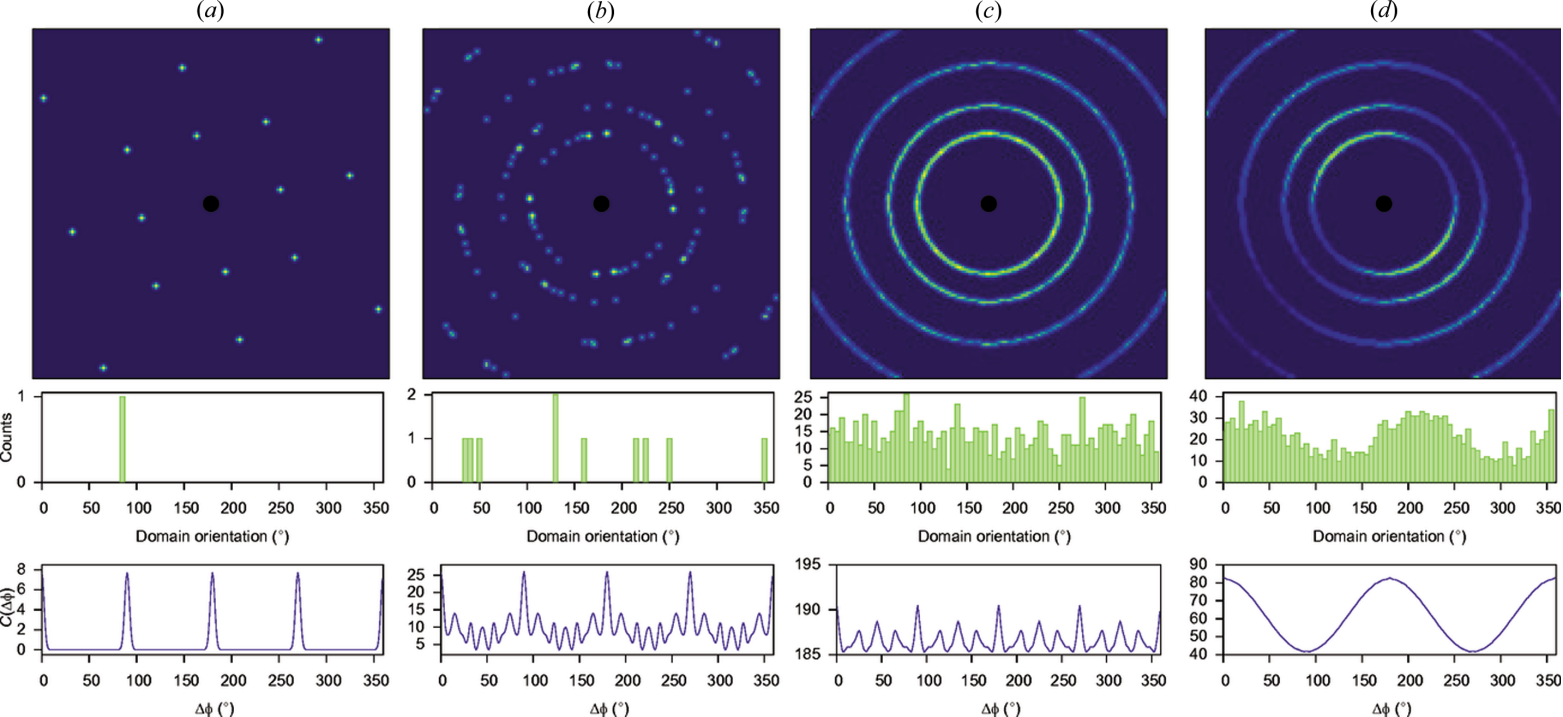
Examples of powder diffraction patterns from (*a*) a perfect single crystal; (*b*) a sample with a small number (*N*_c_ = 10) of domains with uniform pole distribution; (*c*) a sample with a large number (*N*_c_ = 1000) of domains, again with uniform pole distribution; (*d*) a sample with *N*_c_ = 1000 with anisotropic pole distribution. Reproduced with permission from Binns *et al.* (2022 ▸) under a Creative Commons Attribution (CC-BY) Licence.

Diffraction data contain a lot of information that is ‘encoded’ not just in the positions and intensities of the Bragg diffraction maxima. This holds for single crystals, powder diffraction and cases which somehow lie in between, such as dynamic, disordered or defect single crystals or non-statistical powders (Table 1 ▸). Powder diffraction can give information not only about crystal structure and composition but also on domain/particle size, preferred orientation and degree of crystallinity (Ribárik & Ungár, 2010 ▸). This information can be much more reliably obtained if a 2D pattern is available (Fig. 2 ▸). 2D patterns are invaluable when a sample belongs to a ‘gray zone’, *i.e*. a continuum of different levels of crystallinity that exists between the ‘good powders’ and ‘good single crystals’, as is often the case for materials [see *e.g*. McMahon (2004 ▸)]. In particular, some ‘poor powders’ can be treated as a collection of many single-crystalline grains and processed using the tools of multi-grain crystallography. This also applies for samples at high pressure inside a diamond anvil cell (DAC), after laser heating or sintering [see *e.g.* Rosa *et al.* (2015 ▸), Zhang *et al.* (2019 ▸), Aslandukov *et al.* (2022 ▸) and Ledoux *et al.* (2023 ▸)].

**Figure 3:**
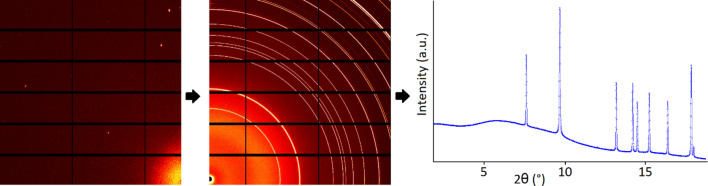
From left to right, a schematic representation of a single-crystal image preserving all 3D information, a 2D powder diffraction image and a powder diffractogram derived from a 2D frame after integration.

At the same time, as compared to single-crystal diffraction, quite a lot of information is lost in powder patterns (Fig. 3 ▸). Multiple images (frames) and several hundreds (if not thousands) of reflections that form a 3D pattern are collected in a single-crystal diffraction measurement, making analysis and integration usually a robust process. Instead, in a powder diffraction measurement, a single 2D frame is collected with a 2D detector, or a linear ‘*I* versus θ’ pattern with a point detector. Contrary to a single-crystal diffraction experiment, the reflections with different *hkl* cannot be resolved if they correspond to the same *d* value. Therefore, indexing of reflections and finding cell parameters and crystal symmetry is not a trivial task.

Symmetry of the crystal structure that is quite obvious in 2D single-crystal diffraction patterns is not so easily revealed when examining 2D powder patterns, and even less so when only a linear ‘*I* versus θ’ pattern is available. The same is true for modulated structures, twins, or structures with static or dynamic disorder.

However, powder diffraction data usually work well for measurements/experiments requiring statistical analysis, *e.g*. quantification.

The major problems – which seem to be not so common for single-crystal diffraction data – are that fitting and refinement of data are prone to end at false minima, and that often one has to go back and look again at raw data, sometimes after many years. The data should then not simply be a sequence of angles and intensities but contain full profiles and include all aspects necessary to interpretation (metadata). The good news is that, containing smaller sized datasets as compared with single-crystal ones, powder data also normally require less space for storage.

## 3. ‘Rare’, ‘medium’ or ‘well done’ – how raw do the deposited raw data need to be?

Although the necessity of preserving raw data is emphasized quite often, it remains disputable how raw the deposited data need to be. Gérard Bricogne raised this problem in his talk at the Melbourne CommData Workshop at the XXVI IUCr Congress, Melbourne, Australia, August 2023 (Bricogne *et al.*, 2023 ▸). In this contribution, we add our thoughts to the discussion.

As has been emphasized by several authors [see *e.g.* Aranda (2018 ▸)], ‘raw data’ is a very difficult term to define. For example, the ICDD defines raw as ‘true’ data, that were not ‘smoothed, α_2_ stripped, background subtracted, or subjected to any other process that would cause significant changes in the true experimental data’ (https://www.icdd.com/grants/acrobat/grntapp.pdf). The 10000 ‘raw powder diffraction datasets’ hosted by the ICDD are in fact these types of data. This is, however, not a universal definition, and many authors understand differently which data should be termed ‘raw’. This is a subject of numerous discussions at different levels. These discussions are not scholastic – depending on what one understands under ‘raw’, different amounts of data in different formats need to be deposited.

Actually, even the first dataset store out of a detector (normally termed ‘primary data’) can be already processed by the firmware of the detectors. These ‘primary data’ can be corrected and then *pre-processed* and *post-processed* to give the final ‘ready-to-analyze scientific data’. These are termed ‘reduced data’, which points to their simplified nature, their improved usability and their usually reduced size for storage. These reduced data are further used in order to obtain what are called ‘derived data’ and what are usually reported in the scientific journals (Table 2 ▸). If raw data are in fact several (related) datasets (for instance, primary, pre-processed, post-processed), a key question arises: which ‘raw data’ should be shared by a meticulous researcher? There is no community-agreed answer (Aranda, 2018 ▸).

If raw data are deposited, it becomes necessary to deposit also ‘metadata’. Metadata are attributes that are necessary to locate, fully characterize and ultimately reproduce other attributes that are identified as data. The metadata include a clear and unambiguous description of the data as well as their full provenance. So, metadata are (Ghiringhelli *et al.*, 2023 ▸) ‘structured information that describes, explains, locates, or otherwise makes it easier to retrieve, use, or manage an information resource. Metadata is often called data about information, or information about information.’

When the importance of depositing and storing ‘raw data’ is emphasized, what is sometimes in fact meant is making ‘reduced data’ accessible. For example, for many years it was supposed sufficient to save ‘relative intensities versus θ’ or ‘relative intensities versus *d*_*hkl*_’ for powder diffraction data and lists of structure factors 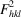 for single-crystal data, in addition to unit-cell parameters and atomic coordinates. Already here we encounter a different level of processing data before depositing them. Relative intensities versus θ are measured directly; the corresponding *d* values can be calculated in a straightforward way, but assigning *hkl* indices to each *d* value in order to calculate lattice parameters requires a special procedure, which can, in general, give not unambiguous or erroneous results. Structure factors depend even more on how data were processed, and atomic coordinates are most sensitive to the selected refinement procedure and the choice of the space symmetry group and the model.

In many cases the algorithms of data reduction are well established and routine, and it may be in fact appropriate to save resources and deposit the reduced data only (Aranda, 2018 ▸). In many other cases, however, obtaining derived data is related to applying various corrections, masking, imposing constraints and restraints, and selecting one of many possible strategies. Quite often, this is done not automatically but manually, so that the role of the ‘human factor’ cannot be neglected. The role of the ‘cook and kitchen’ is here also very important, not only because of the mentioned human factor but also as less complicated measurements, say from a traditional Bragg–Brentano diffractometer in a home laboratory, may require a different approach to more sophisticated ones, such as an *operando* experiment at a synchrotron beamline. Clearly, the higher the number of measurement parameters, the more metadata there must be. The metadata pertains both to the different sample environments and to the data collection techniques. For example, neutron diffraction studies may require more metadata. Ultimately the ‘bill’ is a relatively important factor, where the cost/benefits of storing more or larger datasets (as mentioned, raw data are typically larger in nature) may save experimentalists from having to repeat a whole experimental chain, which may include synthesis or sample treatment. In some cases, this is also intrinsically not possible, *e.g*. a natural sample may have been disposed of and is simply not available anymore. At synchrotron sources it is now policy that data should be managed and possibly stored. Helliwell *et al.* (2024 ▸) recently reported on the evolution of data policies at photon and neutron central facilities.

There are many cases when the whole diffraction image or the most-raw data must be preserved, not only after being processed but as registered by a detector. This is true *e.g.* for high-pressure data collection, studies of diffuse scattering, the analysis of modulated structures, or when studying twinned, strained, disordered, defect or multi-phase samples.

## 4. What is in my data? The many ingredients

**Figure 4:**
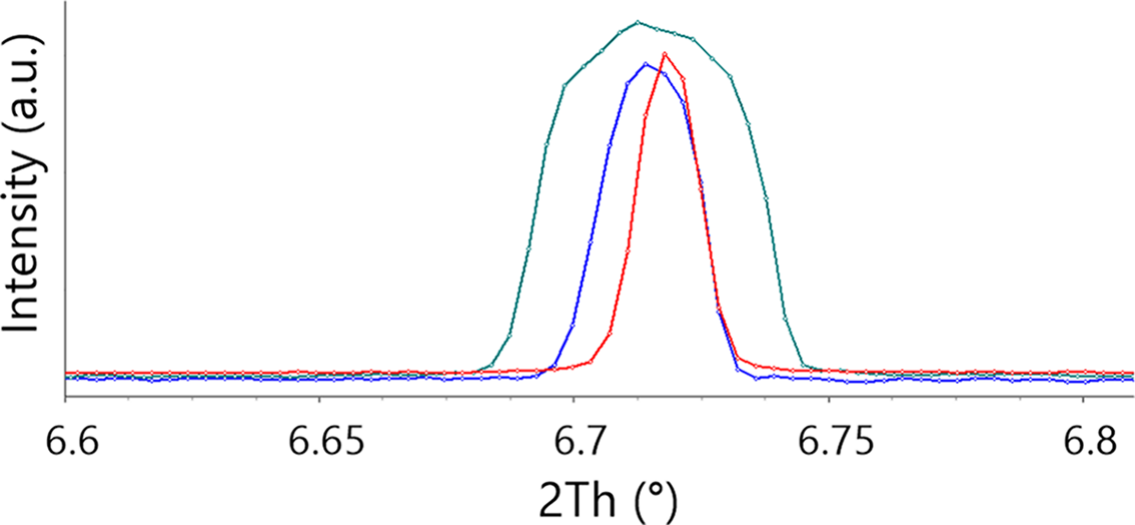
The effect of the sample holder size on the 110 reflection in a powder pattern of fructose: blue – 0.3 mm capillary; green – 0.7 mm capillary; red – 0.7 mm capillary with 0.3 mm slits (slits have a wrong offset).

Temperature, sample origin, instruments used: these are simple and obvious parameters to record and keep. They can and should be controlled. On the other hand, other less obvious parameters may include the sample holder and beam size; they can have a significant effect on the peak width and, in the case of wrong offsets, on the angular position of a diffraction maximum in a powder pattern, as illustrated in Fig. 4 ▸. In this case the cross section between the sample holder and beam size (which may be reduced by slits) defines the geometry of the scattering object that is projected onto the detector. This may produce an apparent broadening and, in the case of a wrong positioning, a peak shift. The wrong positions may lead to wrong conclusions on unit-cell parameters, and wrong peak shapes are related to potential errors in such characteristics of the sample as size and strain of the coherently scattering domains, while also making correct intensity extraction complicated.

Many other things, however, may influence the final pattern, which we cannot fully control *a priori*. In particular, while diffraction measurements are related to the elastically scattered photons, X-rays interact with matter in many ways and this can clearly not be avoided.

## 5. Fluorescence issues

The composition of a sample leading to strong fluorescence (also caused by impurities) may not be known at the beginning and can also change during an *in situ* or *operando* measurement. Fluorescence may, for instance, arise because of the contamination of the sample by steel jar and ball material during mechanical treatment (Boldyreva, 2023 ▸).

**Figure 5:**
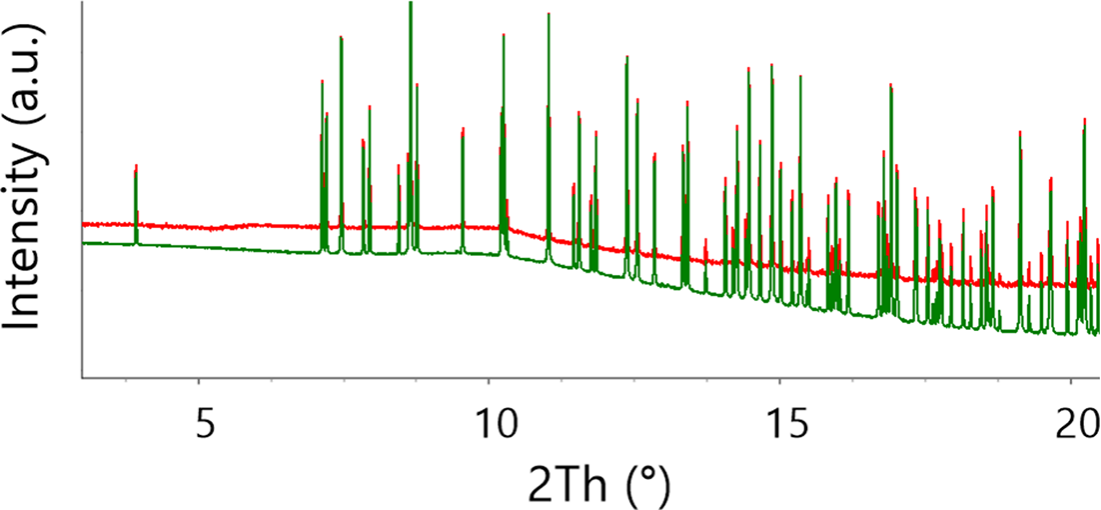
The effect of the fluorescence on similarly collected powder diffraction patterns: green – pattern with fluorescence suppression; red – pattern without fluorescence suppression, including a higher background and non-statistical noise (data taken at the MS beamline, Swiss Light Source).

The effect of the fluorescence on the diffraction pattern can be quite obvious if the background cannot be eliminated. Background correction techniques include the case when single crystals (‘crystal analyzers’) are used in order to select a spectral range from a polychromatic beam or, in some detectors, correctly defining a minimum photon threshold. If not corrected for, fluorescence may lead not only to increased background but to non-statistical noise and other aberrations, which are not so obvious and may result in serious mistakes in interpreting data (Fig. 5 ▸). In particular, non-statistical noise is not accounted for in any refinement software that we know, leading to wrong error estimates, at times wrong intensities and possibly wrong refined parameters. Should the pattern be deposited after the background has been already subtracted, which is in fact often the case in scientific publications, the errors in the intensities might remain unnoticed. Therefore, it is important that information on the detector photon threshold used, the wavelength and the flatfield correction applied, which is also a function of energy, be documented and made available alongside the diffraction pattern itself.

## 6. Background

**Figure 6:**
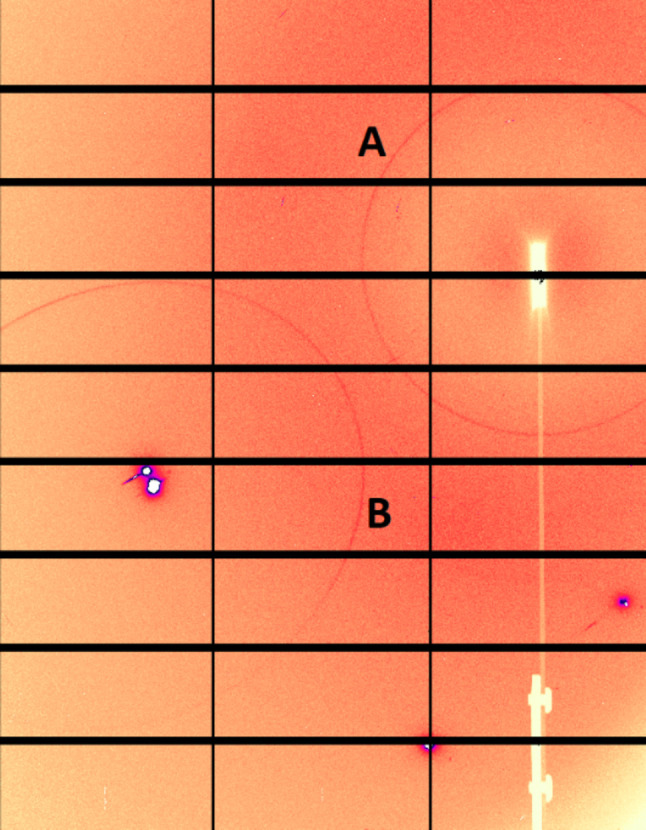
The diffraction ring coming from the primary beam scattering (A) will be correctly integrated, while that from the diamond-diffracted beam (B) will only contribute to the background. The saturating peak at the center of the B ring has to be masked to avoid an extra strong peak in this position.

It is not only fluorescence and diffuse scattering that contribute to the background of the diffraction pattern. The background may contain inelastic, multiple, diffuse scattering which one may struggle to distinguish from the wanted data. There is also Compton scattering and scattered radiation of the sample environment (holder, capillary, reaction chamber, diamond anvil cell, air and so on). In the case of very high intensity data, also the scattering from objects which may be in the diffracted, rather than the primary, beam could end up in the background. In the case of high-pressure data, for example, the beam scattered from the single-crystal diamond may produce diffraction rings from the polycrystalline gasket in the pressure cell, which in a reduced dataset would be lost to the background (Fig. 6 ▸).

Yet again, all this may lead to the wrong extraction of the correct signal, including wrong intensities and wrong evaluation of amorphous content, particularly relevant in the case of less crystalline compounds.

## 7. Peak shapes

As has been already mentioned above, the powder diffraction data are usually deposited in databases and software products in the ‘*I* (integrated or maximum peak intensity) versus θ (or *d*)’ schematic diagram/table format (Fig. 1 ▸). For many applications, however, this information is not sufficient. Obviously enough, a full-profile description is the basis of structure refinement and solution from powder diffraction data (Dinnebier & Billinge, 2008 ▸; Gilmore *et al.*, 2019 ▸). The very first powder diffraction experiments faced the challenge of solving unknown crystal structures, and for some of them (high symmetry, a low number of atoms in the asymmetric unit, all in special crystallographic positions) it was sufficient to know the positions of the diffraction peaks to suggest a structural model (Bragg, 1913 ▸; Bragg & Bragg, 1913 ▸). However, for most cases information on the peak profiles is a must in order to solve a crystal structure.

The analysis of the peak profiles provides information on the size of the coherently diffracting domains (crystallites) and on the lattice strain and stress. Importantly, it is not the shape of a selected single peak or the value of an average peak width that needs to be characterized, but all the peaks in the pattern need to be described. Different types of planar defects may manifest themselves in different broadening of different reflections of the same diffraction pattern. Combined with theoretical modeling, this can shed light on the nature of disorder of the crystal structure. This is key for characterizing minerals, catalysts and various materials (Dinnebier & Billinge, 2008 ▸; Gilmore *et al.*, 2019 ▸). As the interest in nano­materials and nanostructured samples grows, the importance of documenting the peak profiles becomes enormous. Bragg diffraction alone is no longer sufficient to describe a pattern adequately, since diffuse scattering contributes significantly. The theoretical basis known as the Debye approach was developed early on, as described *e.g*. by Guinier (2013 ▸), but its applications only became widespread in recent decades (Tsybulya *et al.*, 2004 ▸).

**Figure 7:**
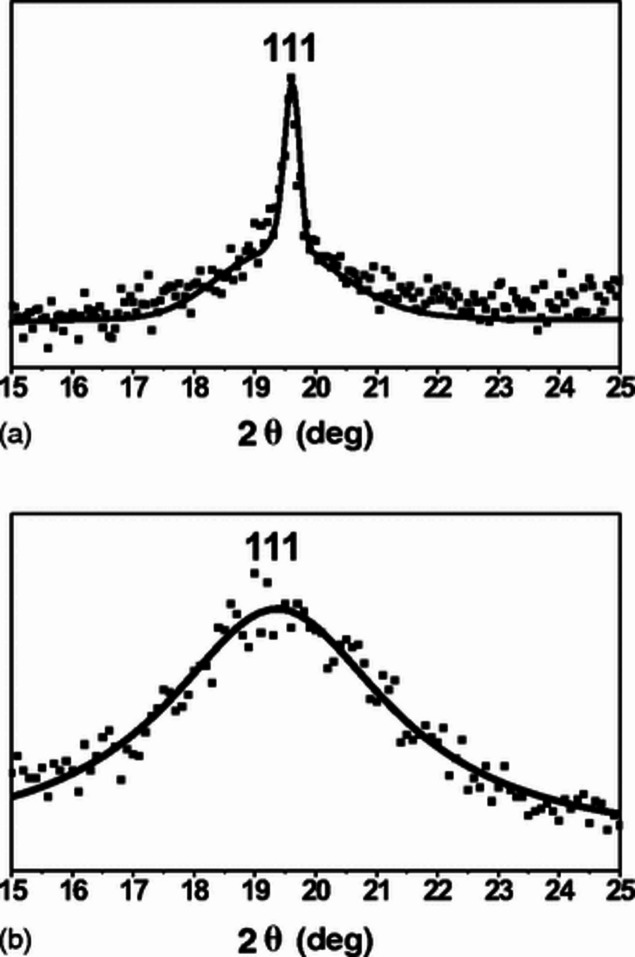
Experimental (dots) and theoretical (bold lines) shapes of the same 111 reflection for (*a*) η-Al_2_O_3_ and (*b*) γ-Al_2_O_3_. In the first case, there are defects lying on a single plane of the {111} family which are bound by the half-dislocation; in the second case, intersecting defects on (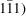 and 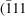) are bounded by the half-dislocations at a dislocation density equal to 20%. Reproduced with permission from Tsybulya & Kryukova (2008 ▸). Copyright (2008) the American Physical Society.

It is generally agreed that full-profile analysis is needed for correct quantitative analysis of the phase composition of multi-phase samples. However, there is still an opinion that peak profiles are not needed for qualitative phase identification (‘fingerprinting’). This is, though, not true for many natural and synthetic materials, some of which are produced by industry in amounts reaching hundreds of thousands of tons per year. As an example, we can mention several different forms of aluminium oxide – the products of low-temperature decomposition of various aluminium hydroxides. Their *I*(*d*) diagrams are practically identical and can be distinguished from each other only if the full profiles of the peaks in a diffraction pattern are available (Fig. 7 ▸) (Tsybulya & Kryukova, 2008 ▸; Busca, 2014 ▸; Rudolph *et al.*, 2019 ▸; Kovarik *et al.*, 2024 ▸). The problem of correctly identifying these phases, which are mass manufactured, is very important for industry, since they are used as catalysts, high-porosity catalyst carriers and precursors in the manufacturing of ceramics. Their properties (in particular, base–acid characteristics) differ radically, and this is critical for their applications.

Another example is related to identifying carbon phases, namely ordered hexagonal graphite (h-graphite) and turbo­stratic carbon (t-carbon), which is widely used in industry as a catalytic material. They cannot be distinguished if only the positions and relative intensities are known. The shapes of the peaks must be analyzed, in particular that of the *hk*0 peaks. Moreover, the diffraction angles and full widths at half maximum (FWHMs) of the diffraction lines cannot be simply used to characterize the lattice parameters and crystallite sizes of t-carbon (Li *et al.*, 2007 ▸). Also some organic polymorphs can be identified reliably only if the full profiles of the powder diffraction peaks are available (Shtukenberg *et al.*, 2017 ▸). The understanding of the importance of preserving full profiles at least for certain classes of materials is growing, so that *e.g.* the PDF database now contains such data at least for materials like cellulose (Kabekkodu & Blanton, 2024 ▸).

**Figure 8:**
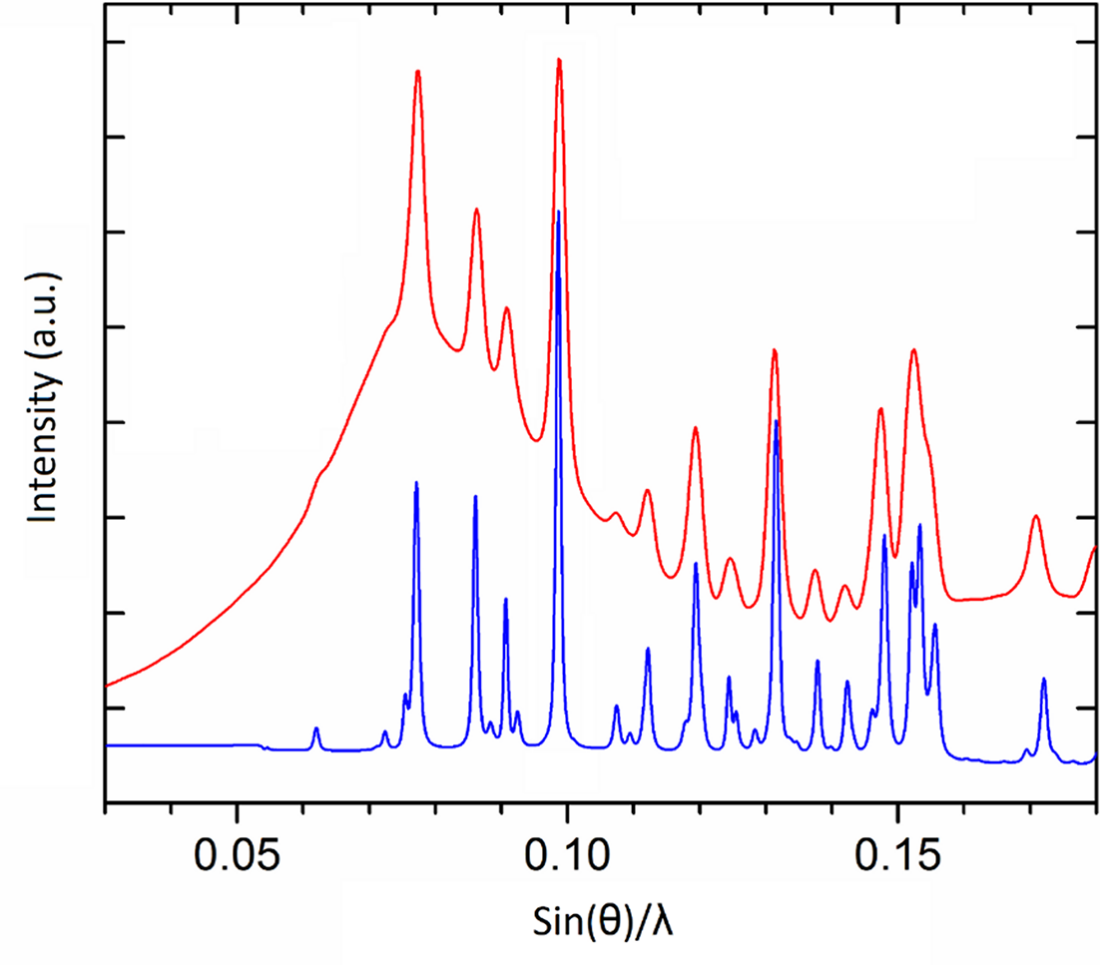
The diffraction patterns collected from the product of the mechanochemical reaction of LiBH_4_ with CsBH_4_ from two different *operando* set-ups: in red that described by Friščić *et al.* (2013 ▸), in blue that by Ban *et al.* (2017 ▸). In the latter pattern, more features are clearly visible due to the increased resolution and smaller background, but it is also clear that the product is the same.

It is also very helpful to have the whole powder diffraction pattern deposited, and not only an *I*(*d*) diagram, when one aims to compare data obtained in different experiments, in order to decide if the experiments gave the same products. As an example we can refer to monitoring a sample in a mechanoreactor during mechanical treatment in real time. Such studies are termed as time-resolved *in situ* (TRIS) experiments, and powder diffraction using synchrotron radiation is one of the important techniques in this field (Michalchuk & Emmerling, 2022 ▸). The early protocols for TRIS powder diffraction gave data of poor quality. The X-ray scattering signals were broad and their shapes were corrupted. This happened because the stationary X-ray beam interacted with a permanently moving powder or slurry within the vessel, while also being absorbed, scattered and diffracted by the walls of the mechanoreactor itself and the moving milling bodies. This is analogous to capturing an image of an object that is not in focus and is permanently changing its location, all while looking through a poorly transparent and light-scattering glass. Moreover, for an inhomogeneous sample moving in the beam (which is usually the case), the composition of the sample can differ at every moment. Different parts of the sample are exposed to the probing beam as the sample moves, and the powder composition changes as the mechanochemical process evolves (Boldyreva, 2022 ▸). Combining innovations in the design of the ball milling apparatus, data acquisition methodology and data processing algorithms, researchers could improve significantly the quality of diffraction data (‘defogging the view’) (Ban *et al.*, 2017 ▸; Michalchuk *et al.*, 2017 ▸; Lampronti *et al.*, 2021 ▸; Mazzeo *et al.*, 2023 ▸). These new developments opened many new possibilities for mechanochemical research. One can now not merely identify the presence of known phases in the reactor and follow the changes in their content but also readily link the effects of mechanical treatment on the particle size and strain with structural and chemical transformations. Moreover, distortions and other subtle structural features that were previously hidden by poor diffraction profiles now became visible. It may be useful to look at previously collected poor data from another experiment after a better pattern could be collected under different conditions, at least to identify the products obtained in the earlier experiment and to compare them with what was observed and characterized later with higher-quality data (Fig. 8 ▸). Availability of full patterns makes conclusions on the similarities or differences in phase composition more reliable. It also becomes more obvious that the broader peaks in an earlier experiment were related not to the characteristics of the particles in the sample but to the imperfect data collection strategy.

## 8. Radiation damage

**Figure 9:**
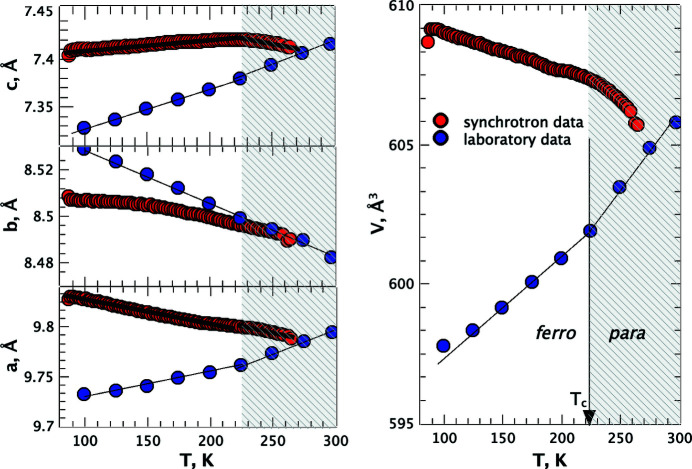
Changes in the unit-cell parameters (left) and volume (right) of glycinium phosphite collected at a synchrotron (red symbols) and a laboratory (blue symbols) source. Reproduced with permission of the International Union of Crystallography from Bogdanov *et al.* (2021 ▸).

Radiation absorption may lead to sample damage. Such sensitivity may not be known in advance and can affect the pattern in long measurements (also in synchrotron ones, especially if repeated at multiple temperatures or pressures). The pattern may look quite normal without any obvious evidence of sample degradation, such as peak broadening, an increased background, the loss of intensities, or a change in the peak intensities and positions. Still, the unit-cell parameters and volume may change because of the radiation damage. These changes may overlay the changes resulting from temperature or pressure variation and in this way corrupt the results of measuring the compressibility or thermal expansion. The data presented as an illustration in Fig. 9 ▸ (Bogdanov *et al.*, 2021 ▸) were obtained at a synchrotron and a laboratory source in single-crystal diffraction experiments, but similar effects can be expected also for long-lasting high-precision powder diffraction experiments, *e.g.* those performed at multiple temperature or pressure points. The sample history and the history of its exposure to X-rays, as well as the dose of radiation obtained by the sample, are therefore important to control and to document. This control and documenting need to include not only the time during which the powder diffraction data were collected but also all the times when the sample was exposed to radiation for alignment purposes, or during other operations in a complex experiment. With recently growing interest in the effect of radiation on the phase transitions in organic (Christensen *et al.*, 2019 ▸; Collings, 2021 ▸; Chernyshov *et al.*, 2022 ▸; Collings & Hanfland, 2022 ▸) and inorganic (Grzechnik *et al.*, 2023 ▸) crystals and in the relation between the anisotropy of thermal and radiation strain (McMonagle *et al.*, 2024 ▸; Boldyreva, 2024 ▸), it would be valuable to have numerous diffraction datasets collected at various synchrotrons accompanied by the information on the radiation dose received by the samples.

## 9. Instrumental resolution function; spinning and rocking of the sample; keeping all images

In many cases data could be of limited value unless the full *instrumental resolution function* (IRF) is known. The IRF is a fundamental characteristic of any diffractometer. It describes the peak profile measured and its angular dependence, due to the specific geometry and conditions of that particular diffractometer. Any diffraction pattern is a convolution of the scattering function from the sample and the instrumental resolution function for the diffractometer on which it was measured. The IRF is needed to extract higher-level information, beyond the simple fingerprinting. The accurate decoupling of these contributions is important for structural and microstructural refinement from Rietveld and pair distribution function analyses of powder diffraction data. The IRF can be routinely characterized with standard, highly crystalline samples and, in many cases, it can also be calculated theoretically or modeled with ray-tracing simulations. It should normally be kept also as metadata or as data from a profile calibrant in the closest possible configuration to the sample (Chernyshov *et al.*, 2024 ▸). Unfortunately, the IRF is often not easily findable when attempting to analyze earlier obtained diffraction data. An experimentalist may not know in advance that this information is relevant, considering fingerprinting sufficient at the moment of collecting data. However, further analysis, such as *e.g*. analysis of coherently diffracting domain size or microstrain in a single domain, is hampered by the lack of a proper IRF.

The* level of spinning or rocking of the sample*, which are measures typically taken to improve the particle statistics and help the diffracted signal from powder to resemble perfect cones, should be recorded. A 2D image is not always available to reevaluate the quality of such particle statistics. When an insufficient number of grains or a heavily oriented sample is present, rocking or spinning is typically employed, when possible, even in the case of large chambers such as a cryostat for high-pressure measurements. The knowledge of any movement, in particular (but not only) in the case of measurements involving a very small amount of sample, may help guide the analysis and decide the level of trust that can be attributed to integrated intensities. Even a Si standard sample, when not appropriately spun, can lead to wrong intensities or wrong peak shapes. In the case of repeated measurements during the monitoring of a process (cooling, dehydration *etc*.) the level of spinning/rocking of the sample may also lead to periodic artifacts within the cascade of data collection, and it is therefore a relevant parameter to later understand the data. Such artifacts may be as puzzling as the periodic appearance of peaks, relative intensity or change in background, which an experimenter would find hard to explain after data processing.

**Figure 10:**
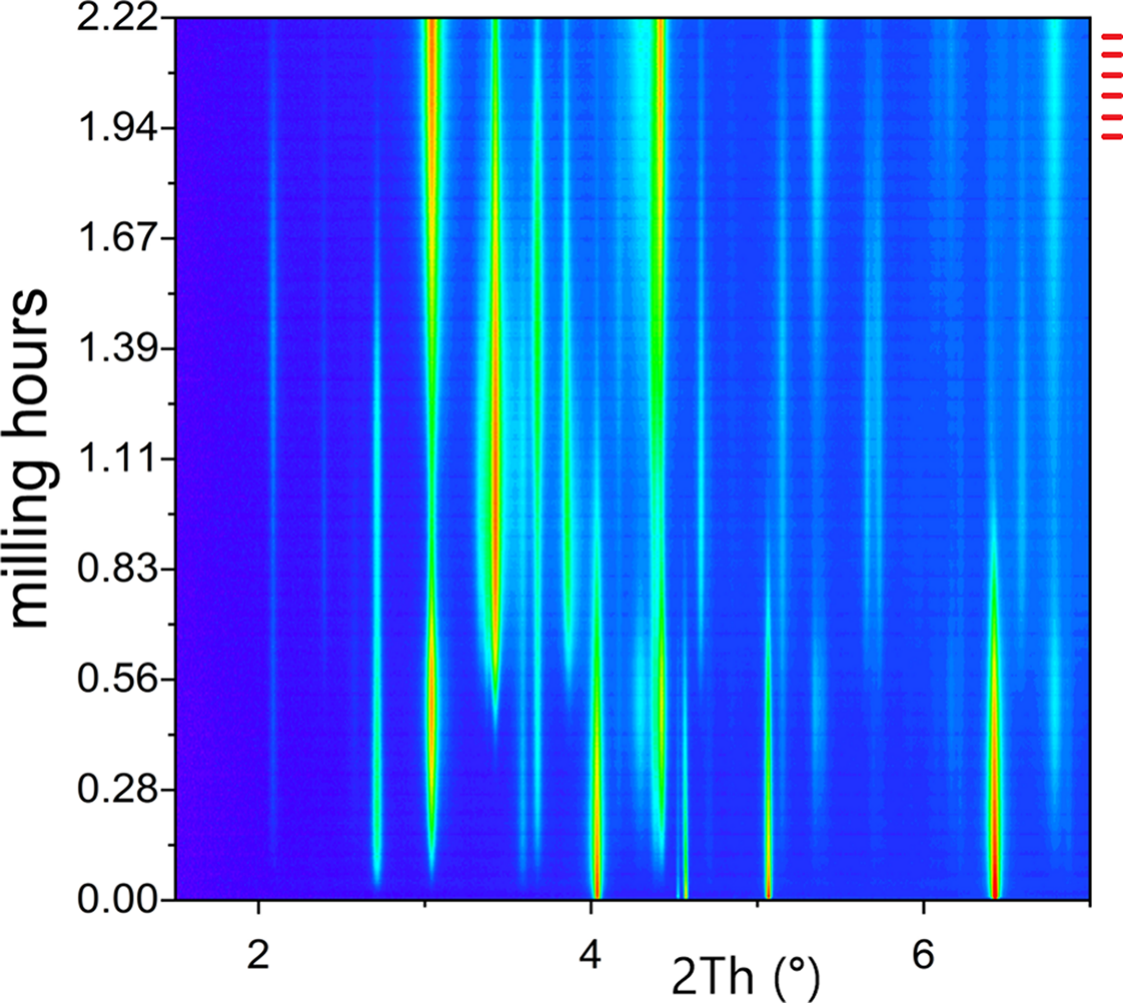
A ‘cascade plot’ of diffractograms from an *operando* diffraction study of a ball-milling reaction. The red marks at the top right indicate points in time where the synchrotron top-up happened.

Time-resolved data often include thousands of images. Tracking changes is relevant, and there is no alternative to *keeping all images* and as many parameters as possible. This is the case, for example, of the data shown in Fig. 10 ▸, coming from an *operando* ball-milling experiment. In the vertical time line it is possible to see the synchrotron top-up, as perturbation (less intensity) every 3 min. This information included into the image is relevant to distinguish this from an effect coming from the milling process itself, such as balls or jar repeatedly finding themselves in the same exact position, which would not guarantee a statistical process and may have suggested the necessity to perform the experiment again (Wilke *et al.*, 2021 ▸). Fortunately, in the example shown, this effect will probably not change the results much, but it is the ability to identify reasons that explain a behavior that is relevant. If data cannot be understood then, in the best case, they are abandoned and, in the worst, very exotic theories arise (or the data may even be massaged to erase difficult observations, which is another big problem).

## 10. Data processing

Area detectors have become the predominant type of detector for the rapid acquisition of X-ray diffraction, small-angle scattering and total scattering data. These detectors record the scattering for a large area, giving each shot good statistical significance to the resulting scattering intensity *I*(*Q*) pattern. Also, the use of area detectors for the entire range of measurements on powders and for total scattering has become widespread because of the temporal advantages. However, although 2D powder diffraction data are much more informative than a 1D diagram, for the analysis and subsequent deposition, powder diffraction data from a detector are usually converted into ‘*I* versus θ’ format. This conversion can lead to errors if calibrations are not perfect or if not all corrections are done properly. While the intensity is associated with the detector performance, the angular part may be linked to a motor, with its intrinsic quality and positional errors, or to an angular conversion (in 1D or 2D position sensitive detectors).

A general trend is to make the conversion, including introducing all the necessary corrections, as automatic and ‘user free’ as possible. At the same time, the less ‘standard’ the experiment is, the more human involvement becomes necessary, and this option is also provided by most popular software (Hinrichsen *et al.*, 2006 ▸; Sims, 2014 ▸; Hammersley, 2016 ▸; Rhymer, 2016 ▸; Vaughan *et al.*, 2025 ▸). Different programs may lead to slightly different processed data and may consider differently spaced outputs (in θ). Corrections and calibrations may have to be revisited for a number of reasons. Therefore, it is very important to preserve the data before any corrections have been introduced, and also a clear description of the corrections to these data and a justification of why these corrections were necessary.

**Figure 11:**
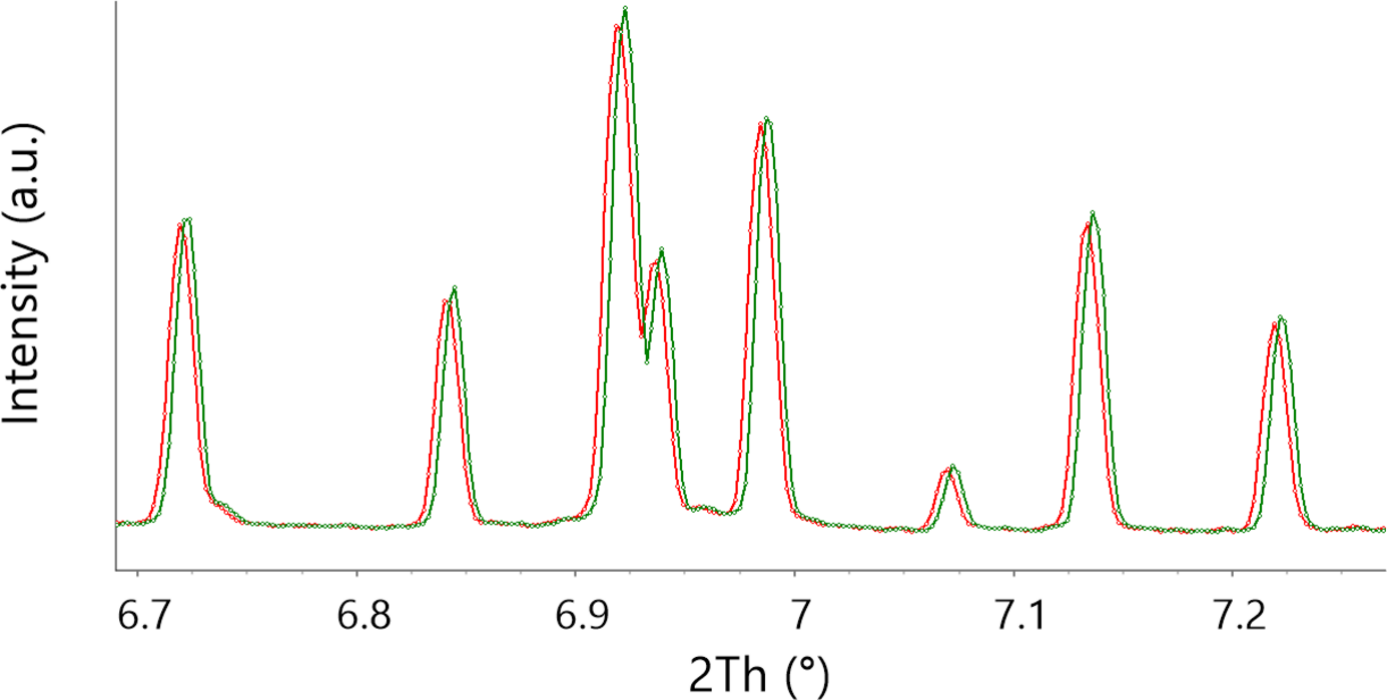
Effect of a wrong calibration on radially integrated peaks, illustrated by an angle-dependent shifted pattern (in red) with respect to the correct values (in green). The errors in the peaks’ positions are non-linear with the angle but of the same order of magnitude as the peaks’ FWHMs. While fingerprinting was correct, the refinement of the patterns was not converging as the peak positions are not simply shifted but affected by a more complex function.

In the example illustrated by Fig. 11 ▸ the 2D → 1D conversion was at first wrong. This was due to the Al_2_O_3_ used for initial calibration, for which the unit cell was slightly wrong because of contamination during synthesis. Data were not immediately refinable, and only after realizing the mistake could a correct model be applied, the correct angular calibration obtained and the data successfully reanalyzed.

**Figure 12:**
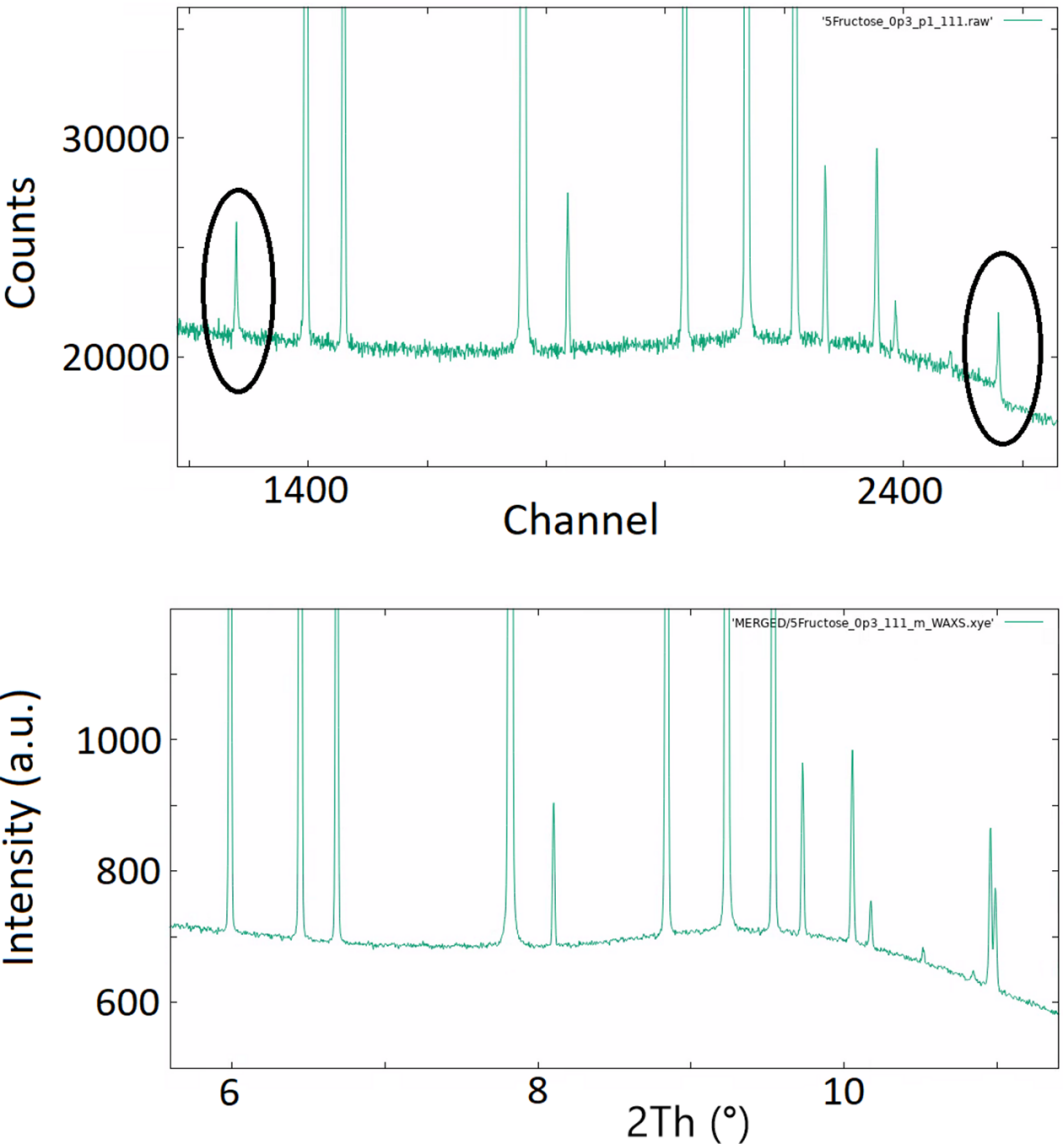
Data from a Mythen detector: the raw data (above) show spurious features, but appropriate correction for flatfield and angular calibration results in the correct pattern (below). The most affected parts of the patterns are encircled. The result of these errors if not corrected would be a noisy, non-statistical background, the presence of spurious features and non-corrected intensities of the peaks, leading to data unusable for structure solution, quantification and even fingerprinting.

Keeping track of how a calibration is performed and original images is therefore relevant to correct potential problems that may be seen only at a later stage. At the same time, before the generation of ‘standard’ patterns, a flatfield correction may be associated with a multichannel detector which has to be revisited at a later time. Also in these cases, both the raw 2D data and information on how the processing has happened are relevant to guide further analysis. For example, the Mythen system employed at several synchrotron facilities requires a flatfield and an angular calibration, while area detectors require a flatfield (in some systems hidden behind the initial output), an angular calibration and a mask for shaded areas. ‘Fake’ features such as incorrect peaks or background ‘jumps’, wrongly positioned peaks, or even peak shapes may be affected by the process (Fig. 12 ▸). Therefore, it is important to preserve data both before and after calibration, flatfield correction and masking.

**Figure 13:**
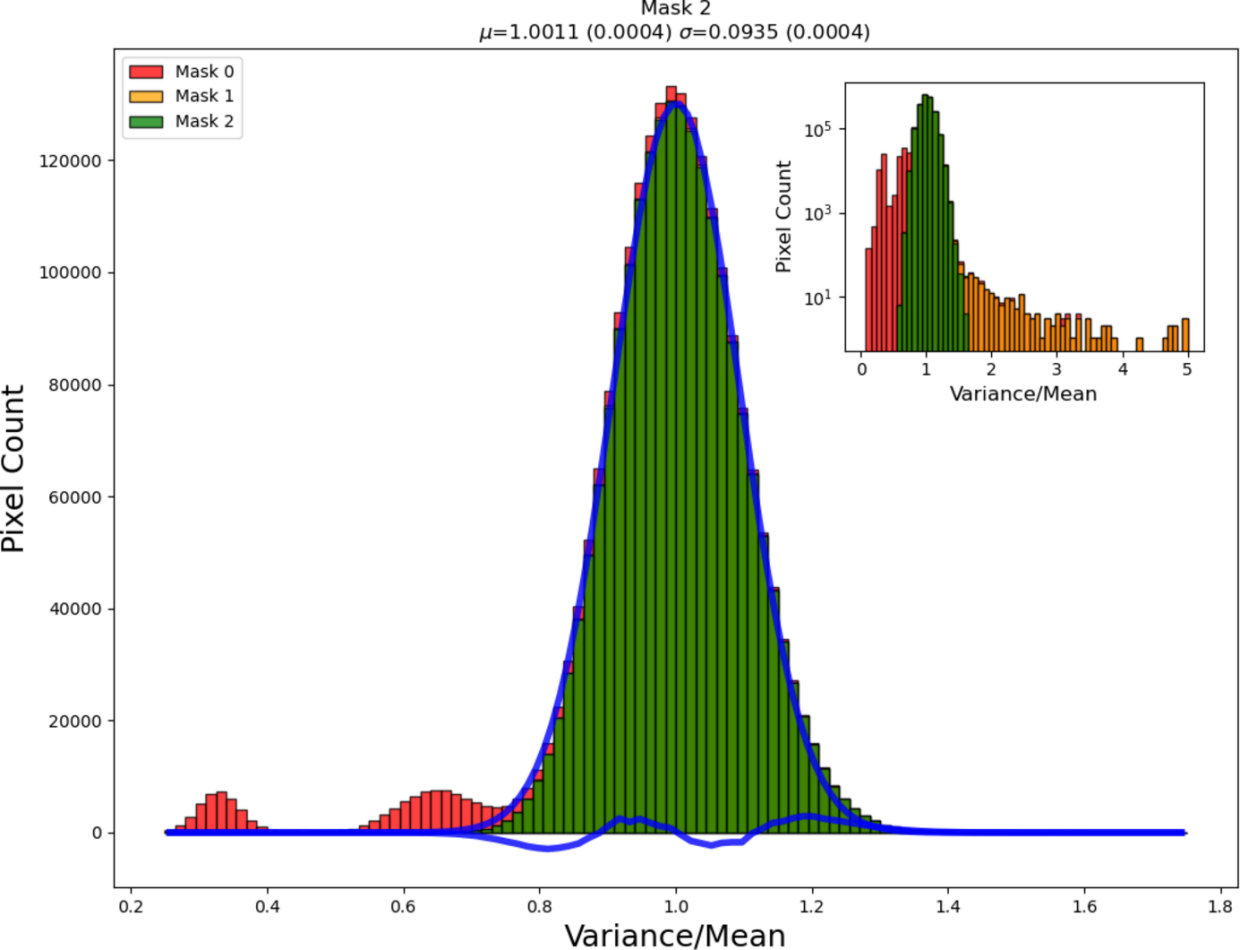
Effect on the counting statistics over the detector of appropriate masking of non-Poissonian and multiple pixels. The distribution of the variance over the mean counts per pixel, measured pixel by pixel for 256 images of silicone oil. Masks 0, 1 and 2 correspond, respectively, to masking only the pixels flagged by DECTRIS, masking also the rebinned pixels between sub-modules and detector edges, and masking also pixels with high σ/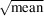 (typically damaged pixels) as well as dilating by 1 pixel the regions around each masked pixel. The percentages of pixels masked in each case were 8.6%, 15.4% and 15.8%, respectively. The blue lines are a Gaussian fit to the final (mask 2) data and the difference curve. Inset: the same data on a semi-log scale. Reproduced with permission from Vaughan *et al.* (2025 ▸) under a Creative Commons Attribution (CC-BY) Licence.

Many detectors (single-photon-counting 1D and 2D detectors, and partially also charge-integrating ones) can be an origin of systematic errors in diffraction data. The errors (*e.g.* those caused by spatial variations in detector efficiency) can only partially be corrected. Pixel-level defects may cause serious errors in the resulting 1D and 2D diffraction patterns. Silicon-based hybrid photon-counting pixel detectors have become common for diffraction experiments of all types at low and moderate X-ray energies. More recently, hybrid pixel detectors with high-*Z* materials have become available, opening up the benefits of this technology for high-energy diffraction experiments. However, detection layers made of high-*Z* materials are less perfect than those made of silicon, so care must be taken to correct the data in order to remove systematic errors in detector response introduced by inhomogeneities in the detection layer, in addition to the variation of the response of the electronics (Vaughan *et al.*, 2025 ▸). Owing to the difficulty in growing CdTe with the same perfection as Si wafers, several pixels on the detector may be defective, exhibiting high noise or non-Poissonian counting, and must be eliminated prior to analysis. An example is provided in Fig. 13 ▸ (Vaughan *et al.*, 2025 ▸). The detector was characterized after fabrication and delivered with a mask file in which these pixels were flagged; they were simply masked during data reduction. Further pixels can become damaged over time (*e.g.* from overexposure), and other pixels can be observed to have unusually high noise (*e.g.* pixels near the gaps and the detector edges) already during use of the detector. It is therefore necessary to periodically check the detector for bad pixels; this can be done while carrying out flood field corrections. The effect of removing the defective and the multiple pixels on the global statistics can be seen in Fig. 13 ▸ (Vaughan *et al.*, 2025 ▸).

Recently developed software (*e.g.* Wright & Zhou, 2017 ▸; Sadri *et al.*, 2022 ▸; Vaughan *et al.*, 2025 ▸) can automatically find and mask the dead pixels and other defects of the detector. If raw data collected using 2D pixel detectors without such corrections (by other groups who do not have this software, or, in general, before the software has become available) could be easily accessed, they could be reprocessed now using this new software with a chance to improve the results.

## 11. *In situ* and *operando* data collection

Diffraction data from a sample often (in fact, more often than not) need additional ‘pre-treatment’ before being used, in order to solve and refine a structural model, to estimate correctly the size of coherently scattering domains, to analyze the defects *etc*. This pre-treatment can include introducing various corrections of intensities of diffraction maxima, background corrections, corrections of the peak profiles taking into account the IRF or exclusion of diffraction maxima not belonging to the part of the sample that is analyzed. Data pre-treatment is a very sensitive issue, since, though unavoidably necessary, it can also introduce errors instead of eliminating them, valuable information may be lost, and the interpretation of the experimental data may be partly or even completely wrong. Various tools have been implemented even into commercial software distributed together with diffractometers, in order to make data pre-treatment as objective and justified as possible, and also to evaluate objectively the data quality before and after having introduced additional corrections. Still, only preserving data as collected, prior to any pre-treatment, can guarantee that any errors, if made, can be found and corrected at a later stage.

Collecting diffraction data *in situ* and under operating conditions adds challenges. Usually, in order to achieve variable temperature and/or pressure conditions, a sample is put in a special thermostat, a cryostat, a high-pressure cell or a chemical reactor (for simplicity, we shall call any of such devices a ‘sample chamber’ in further discussion). As a result, at least three types of problems arise:

(i) Not all the reflections can be observed in a diffraction pattern, since either the incident beam cannot reach the sample or the diffracted beam cannot reach the detector, being absorbed completely by the sample chamber materials (Katrusiak, 2004*a* ▸; Katrusiak, 2004*b* ▸; Katrusiak, 2008 ▸; Katrusiak, 2019 ▸; Dawson *et al.*, 2004 ▸; Angel & Gonzalez-Platas, 2013 ▸; Prescher & Prakapenka, 2015 ▸).

(ii) The intensities of the diffraction maxima can be corrupted because of the partial absorption of the incident and/or diffracted beam by the sample chamber materials or by various external devices (like laser heating optics); also the effects of preferred orientation of crystallites may be more pronounced and more difficult to avoid, since the choice of variants of sample spinning or rocking is restricted by the chamber cell (Katrusiak, 2004*a* ▸; Katrusiak, 2004*b* ▸; Katrusiak, 2008 ▸; Katrusiak, 2019 ▸ Angel & Gonzalez-Platas, 2013 ▸; Stan *et al.*, 2018 ▸).

(iii) Diffraction patterns can be contaminated by various features originating from the interaction of the incident and/or diffracted beam with the sample chamber materials, as well as other phases present in the sample chamber, for example, as medium or calibrants. These can be point reflections from single-crystalline phases, diffraction rings from polycrystals or distorted background (often with a pronounced halo) from amorphous glassy components, as well as various artifacts like pseudo-Kossel lines for data collected in a diamond anvil pressure cell (Katrusiak, 2008 ▸; Katrusiak, 2019 ▸).

The first two problems can be solved if exact information about the geometry of the sample chamber, the sample and the experimental environment is known, and the paths of the incident and diffracted beams can be calculated (Katrusiak, 2004*a* ▸; Katrusiak, 2004*b* ▸; Katrusiak, 2019 ▸; Angel & Gonzalez-Platas, 2013 ▸). These metadata need to be deposited alongside the diffraction data themselves. It is also necessary to document the procedure of implementing shadowing and absorption correction and the software used. The limited access to the reciprocal space makes the datasets collected from a sample in a sample chamber unavoidably systematically incomplete, since a significant part of the incident and diffracted radiation is absorbed by the material of the sample chamber (for example, a DAC). It is not just the weaker and high-angle reflections that are missing but complete sections of the reciprocal space – which sections exactly depends on the orientation of the crystal(lite) with respect to the X-ray beam (Katrusiak, 2008 ▸). For single-crystal diffraction this issue is taken into account by documenting the orientation of the chamber with respect to the beam, which can be achieved by taking a photograph of the crystal in the cell and also by documenting the dimensions of the crystal and the geometrical parameters of the sample chamber (Katrusiak, 2004*a* ▸; Katrusiak, 2004*b* ▸; Katrusiak, 2008 ▸). For powder diffraction, the orientation of the crystallites in a polycrystalline sample cannot be observed directly, but it manifests itself in the distribution of the peaks’ relative intensities, which can be corrupted additionally if a part of the incident and diffracted radiation is screened by the DAC material. Knowing information about the data before having any intensities corrected, as well as the metadata and the software used for these corrections, is important for potentially revisiting the data, if a doubt in the accuracy and correctness of the processed data arises.

Excluding part of the diffraction pattern from consideration (‘masking’) is probably the most sensitive procedure that requires much attention (Dawson *et al.*, 2004 ▸; Hinrichsen *et al.*, 2006 ▸; Casati *et al.*, 2007 ▸; Jensen *et al.*, 2010 ▸; Wilkinson *et al.*, 2011 ▸; Dera *et al.*, 2013 ▸; Sims, 2014 ▸; Prescher & Prakapenka, 2015 ▸; Hammersley, 2016 ▸; Rhymer, 2016 ▸; Casati *et al.*, 2017 ▸; Wright & Zhou, 2017 ▸; Stan *et al.*, 2018 ▸; Katrusiak, 2019 ▸). Masks are applied to particular areas, which are either shadowed or contain diffraction data scattered from sources other than the sample. For example, when data are collected from a sample in a DAC, data masking is not just ‘desirable’ but very often compulsory. Otherwise, the pattern will be corrupted, being ‘contaminated’ by strong point diamond reflections, reflections from single-crystalline or powder pressure calibrants, and, in the case of a large beam size and a small sample chamber, the powder rings originating from the gasket. Sometimes, the hydrostatic medium solidifies and contributes not only to background but also to the diffraction pattern itself. A pattern containing even just a few ‘foreign’ strong reflections may be completely non-interpretable, or its interpretation will give wrong results. At the same time, masking must be justified and documented, so that it does not become an irreversible and non-controlled data manipulation. A mere explicit statement that ‘masking was performed’ (which is always made in a publication) is useless and not sufficient if there is no access to data before such a correction and to the software used for corrections. Masks should be stored together with the processed data and made available as metadata used in the raw data processing procedure.

When collecting single-crystal data, it may be more obvious which part of the diffraction pattern is not originating from the sample. For example, powder diffraction rings originating from the sample chamber polycrystalline material look like natural candidates for masking if the general diffraction pattern consists of point reflections. Such contaminating rings may be excluded experimentally if the diffraction beam is small enough not to hit anything but the sample. This can be achieved easily at synchrotrons but is very rarely, if at all, possible in a laboratory. Also the polycrystalline chamber windows through which a beam penetrates, or Be backing plates of some types of high-pressure DACs, can give these rings contaminating diffraction patterns, and these can be recognized easily and masked. If a sample chamber contains single-crystalline materials (*e.g.* diamonds or ruby calibrants in a DAC pressure cell), their reflections can also be recognized and masked, to exclude them from the dataset used for structure solution and refinement and also to avoid errors in analyzing the background. This processing can today be done completely or partly automatically with the software packages that implement shadowing and absorption correction, as well as masking. These options are also included in the software for data reduction and refinement provided with diffractometers or distributed independently free of charge (such as *e.g.CrysAlis^Pro^*; https://rigaku.com/products/crystallography/x-ray-diffraction/crysalispro).

In the case of analyzing a powder pattern, it is, conversely, more straightforward to mask point reflections from single-crystalline ‘foreign’ sample environment materials but more ambiguous to separate and mask those from polycrystalline pattern-contaminating materials, such as gasket, sample chamber walls, DAC backing plates, powder calibrants or solidified pressure-transmitting fluid. There is software that makes it possible to mask point reflections from single-crystalline ruby and diamonds (Hammersley, 2016 ▸; Prescher & Prakapenka, 2015 ▸). Still, even when using this software, masking is often done manually and is thus subject to a ‘human factor’. For example, if all the point reflections are masked in a powder diffraction pattern consisting of rings, one cannot exclude the situation when reflections belonging to a new phase appearing as single crystals or as large crystallites are also masked and thus overlooked. Point reflections located at rings might belong to a new phase that crystallizes, say, at high pressure. This needs further analysis, before these reflections may be masked. It is advisable therefore to save both original ‘raw’ data and the mask, so that it is possible to improve masking if necessary in the future. Since detectors evolve and so does the software used for processing data, including masking, when depositing data for long-term storage the software suitable for processing these data must also be stored. It is important to perform any masking before the 2D → 1D conversion, since any decision on which reflections do not belong to the pattern or need correction is much less ambiguous in the case of a 1D *I*(2θ) diffraction pattern.

**Figure 14:**
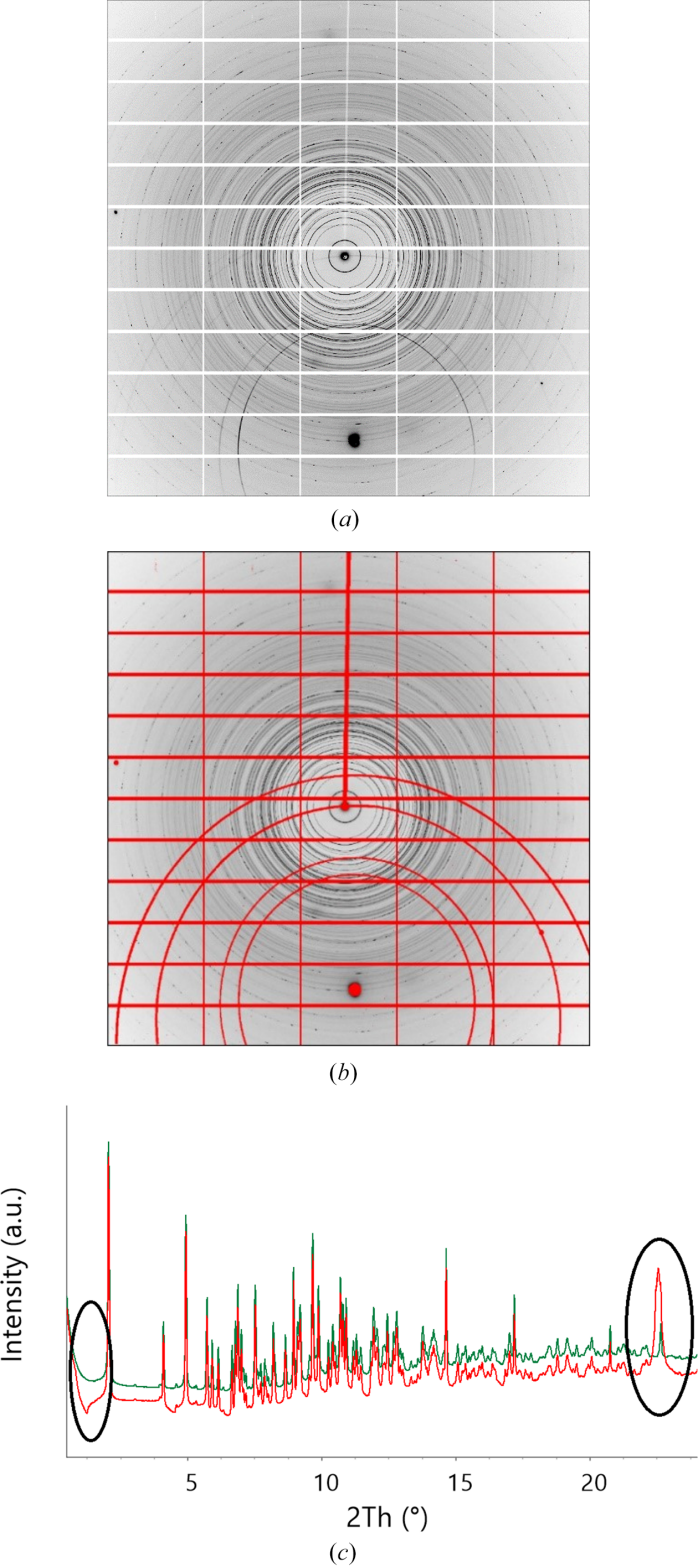
Examples of a 2D powder diffraction pattern collected for a powder sample in a diamond anvil cell *in situ* at a synchrotron source using a 2D pixel detector, with the reflections from diamonds and ruby calibrant (points) overlaying the diffraction pattern from a powder sample (rings): (*a*) before masking, (*b*) after partial masking, (*c*) after 2D → 1D integration without masking (red) and after masking (green). The major differences between the masked and non-masked 1D patterns are circled. The background was not subtracted, but one plot was shifted vertically with respect to another.

Synchrotron data are usually much ‘cleaner’ than laboratory data, since the small beam does not hit the gasket. Still, it is not possible to avoid masking completely, since a single diamond reflection may distort the pattern after 2D → 1D transformation very significantly (Fig. 14 ▸). In addition, the rings around this diamond reflection need to be masked, in order to have correct background (see also Fig. 6 ▸). Owing to the modular nature of the detector, gaps exist between the different modules, and these must be also masked during data treatment. If the primary data were not corrected in the original study, this may be corrected at a later stage, if a higher quality of data processing is needed.

**Figure 15:**
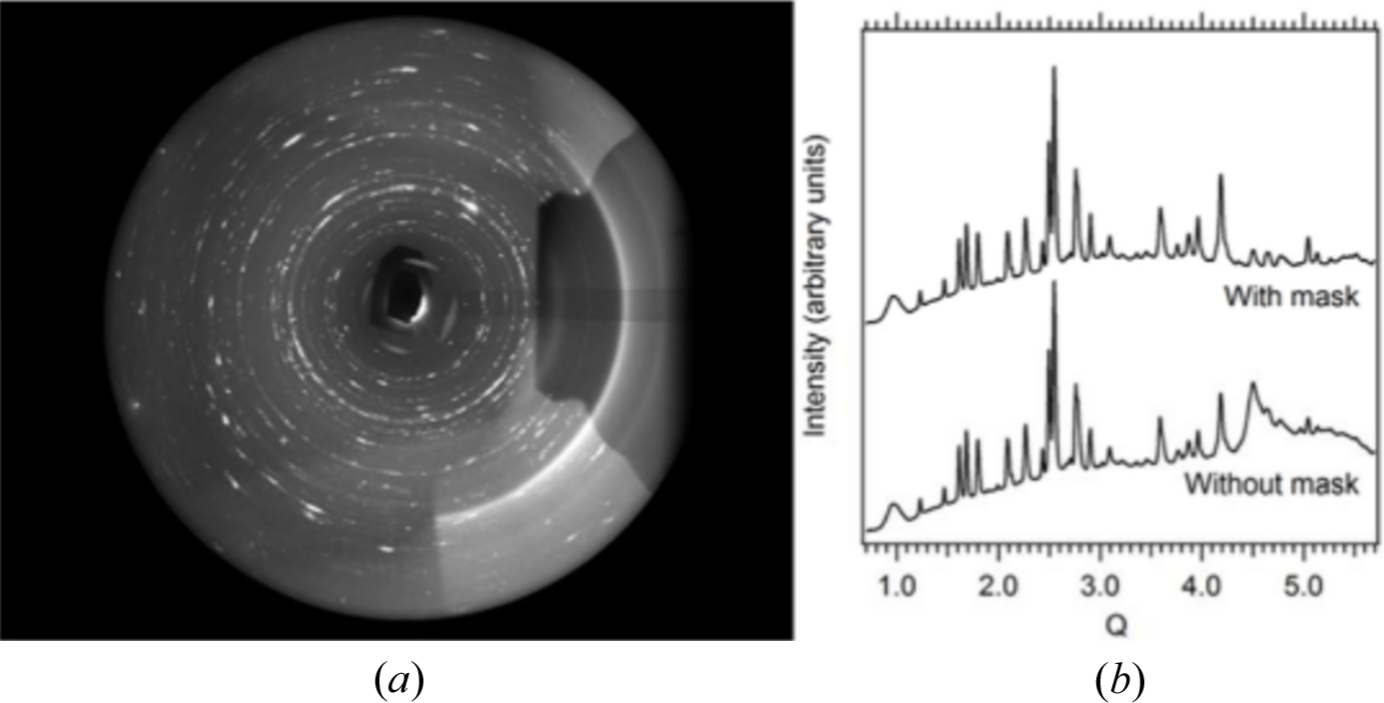
(*a*) A typical raw high-pressure (10 GPa) X-ray powder diffraction pattern (San Carlos olivine) collected during laser heating to 2000 K. The shadow on the right-hand side is caused by the downstream laser heating optics. The broad smooth partial rings on the right side stem from X-ray scattering on the laser mirror. Both artifacts need to be masked out for data reduction and analysis. (*b*) Integrated diffraction pattern with and without masking of laser heating artifacts. Reproduced with permission under a Creative Commons Attribution (CC-BY) Licence from Stan *et al.* (2018 ▸).

In high-pressure high-temperature experiments the heating equipment may also cause shadowing of the powder diffraction patterns that requires masking (Fig. 15 ▸).

**Figure 16:**
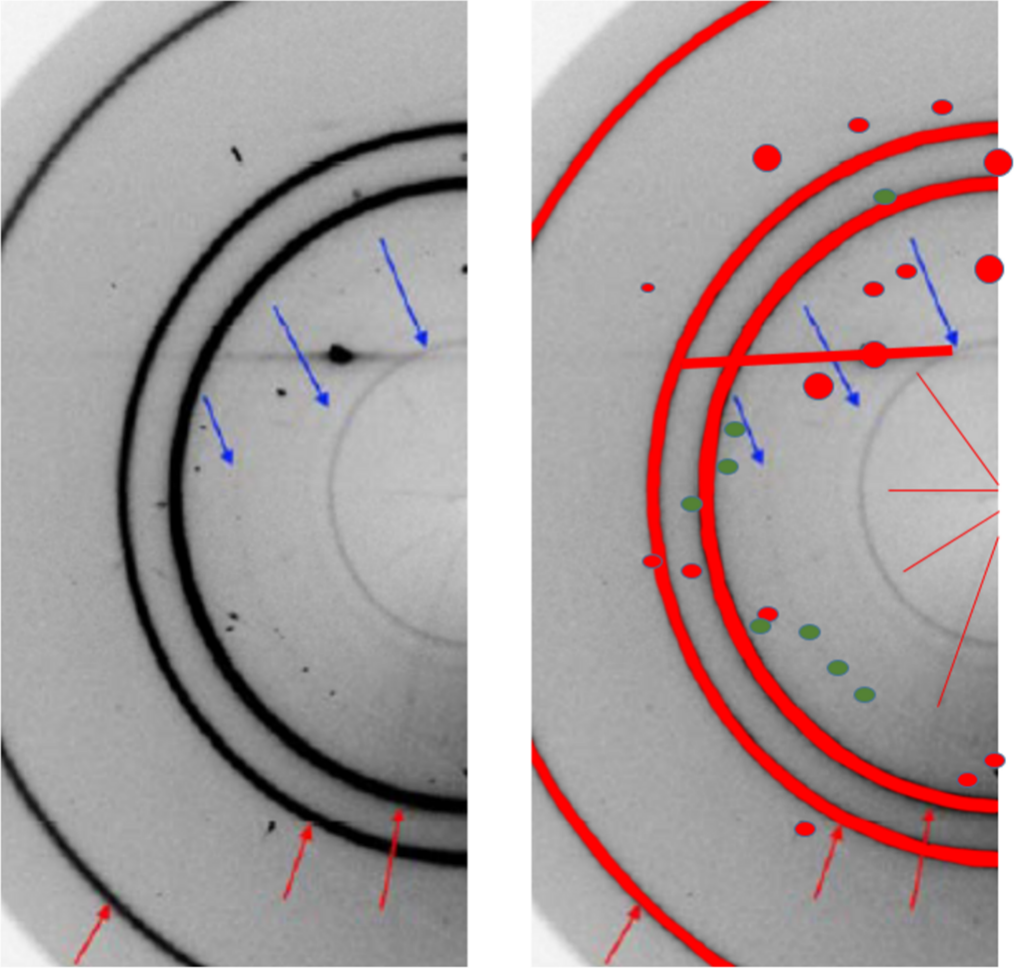
Examples of a 2D powder diffraction pattern collected with a laboratory diffractometer, when the beam size is so large that the beam hits also the metal gasket. One can see the reflections from diamonds, ruby calibrant (points) and steel gasket (bold thick rings shown by red arrows) overlaying very weak rings originating from the poorly scattering powder sample (shown by blue arrows): (*a*) before masking, (*b*) after masking. The red mask is unambiguously justified. At least some of the point reflections of low intensity that were preliminarily masked (green) are located at rings and could potentially belong to a new phase that crystallizes under high-pressure conditions in the DAC. Therefore, their masking is not unambiguously justified; the origin of these reflections deserves further investigation.

For powder data obtained with a laboratory diffractometer, much more pronounced masking may become necessary (Fig. 16 ▸), and the consequences of ‘non-masking’ may be much more serious (the pattern originating from the sample may be completely ‘hidden’ in the background, with much stronger reflections from the gasket dominating).

**Figure 17:**
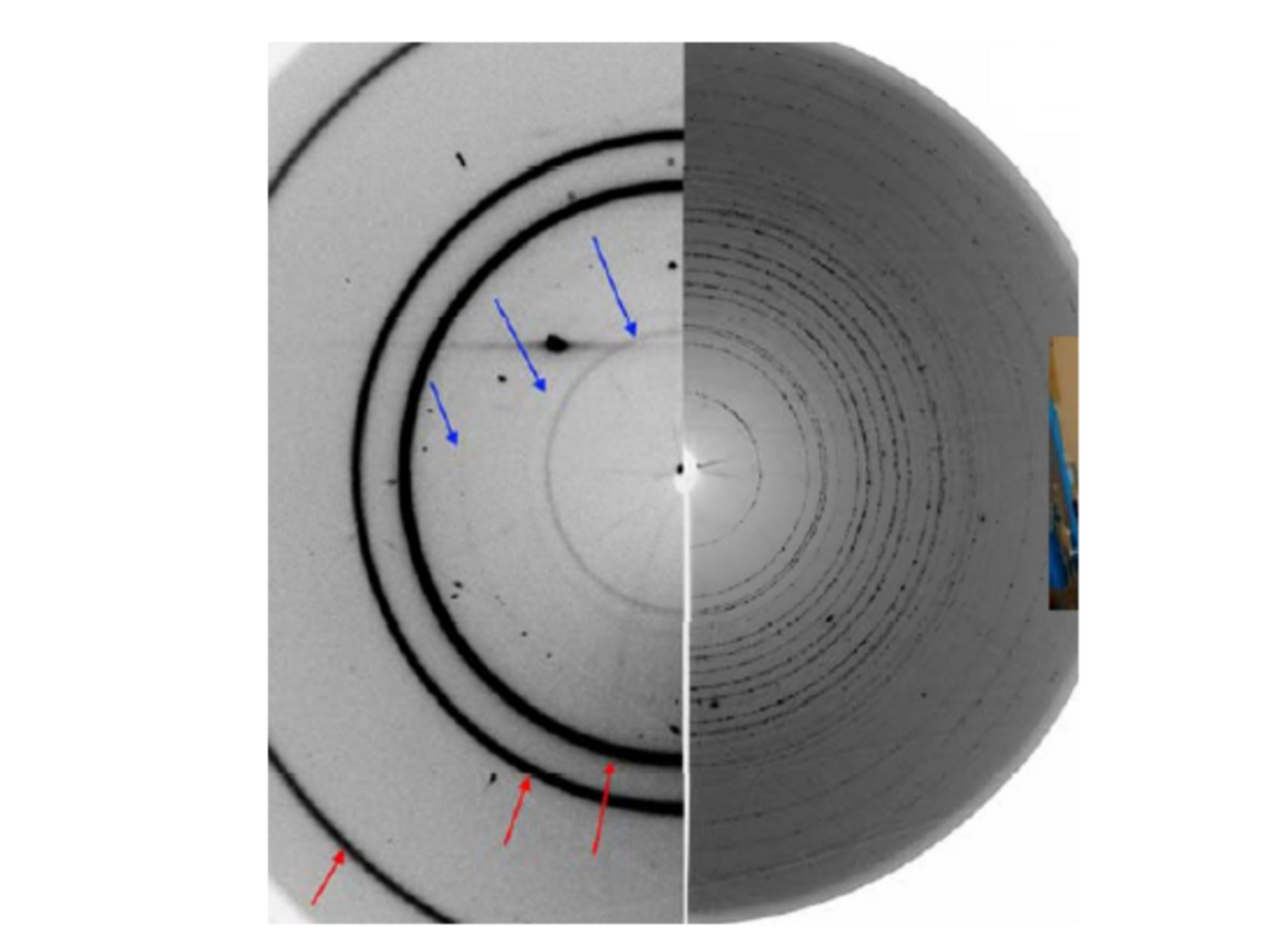
Two powder diffraction patterns (unmasked) collected from the same phase in a DAC at a laboratory source (left) and at a synchrotron source (right). Comparing the primary 2D patterns makes it possible to see that the phase studied in the laboratory is in fact the same as that studied later at the synchrotron source (sample rings in the left photograph are shown by blue arrows). The pattern on the left is the same as was shown in Fig. 16 ▸. Note that masking of the gasket rings (shown by red arrows) was no longer necessary for data collected at the synchrotron source, but masking of a few point reflections originating from diamond would still be needed. One can also see from the comparison that one of the gasket broad bold rings in fact overlaps with two rings from the sample that became accessible in a synchrotron experiment. Data courtesy of Dr N. Tumanov.

The data quality obtained in the experiment illustrated in Fig. 16 ▸ was not sufficient to solve the structure of the high-pressure phase: the diffraction rings that did not belong to the gasket material were too few and too weak. It was also not possible to exclude that some diffraction rings from the sample could be overlaid by those originating from the gasket. Still, it was possible to conclude that the high-pressure phase was a new phase different from the previously known ambient-pressure polymorph. This was sufficient to justify taking this sample to a synchrotron, where much better data could be collected that made it possible to solve the crystal structure. Since the unmasked 2D patterns collected with the laboratory diffractometer were preserved, revisiting these data allowed them to be compared with the synchrotron data to prove that the phase studied previously in the laboratory was the same as the one that was studied later at the synchrotron source (Fig. 17 ▸) (Tumanov *et al.*, 2010 ▸).

Another issue to consider is masking in datasets consisting of series of multiple patterns. The prompt processing of hundreds or thousands of images immediately after a single experiment is crucial for the success of the experimental program. As processing such a substantial amount of data manually in a reasonable time is impractical and sometimes impossible, the challenge can be met by developing automatic pipelines to simplify the process of data analysis and decision making during the experiment. In the case of *in situ*/*operando* data collection, different masks may be required for different images even from the same series. This adds complexity to the development of an automatic procedure and also to storing ‘raw’ data together with a corresponding mask. A recent example of a software package that has been developed at the Extreme Conditions Beamline P02.2 of Petra III – and can be used both on-site and off-site at other beamlines at PETRA III, as well as at other large-scale facilities – was described recently by Karnevskiy *et al.* (2024 ▸). The software was originally developed to be used for dynamic compression experiments employing DACs, enabling experiments over a wide range of pressures and temperatures and providing access to small or medium strain rates inaccessible in shock/ramp compression studies. The overall duration of a dynamic compression X-ray diffraction experiment can be from less than a second to many minutes for acquisition of image sequences. Correspondingly, the final number of signal-bearing 2D detector frames is typically large, ranging from several hundred to tens of thousands of images, collected, for example, along a tunable compression ramp. The developed software could be readily adapted to provide rapid feedback for many other X-ray diffraction techniques, *e.g.* large-volume press studies, *in situ* stress/strain studies, phase transformation studies, chemical reactions studied with high-resolution diffraction *etc*. (Karnevskiy *et al.*, 2024 ▸). More importantly, both ‘raw’ and the finally processed data are stored for future use and potential reevaluation, as well as the masks imposed on the data together with the software package that can be used for data reprocessing, if needed.

## 12. Keeping sample photographs

It is advisable to keep not only diffraction frames but often also photographs of the sample itself. In single-crystal diffraction it is a common practice to take photographs of the crystal in a holder or in a sample chamber and to include them in publications. This visual information may help to identify a polymorph in future studies. It is necessary for numerical absorption and shadowing corrections. Even the color of the sample may indicate potential errors in solving the crystal structure (Massa, 2004 ▸).

For powder samples, taking photographs of the samples is not so common. In fact, it is not always very informative. Still, even merely documenting the color, the shininess *etc.* may help to identify the phase if some doubts arise in the future. Knowing the shape of small particles in a polycrystalline sample may help to identify the phases, to rationalize the corrections for preferred orientation effects or to indicate the presence of impurities, even if they are not detectable in the diffraction pattern (for example, amorphous phases). Taking and preserving photographs and sketches becomes very important when studying natural minerals, composite materials or non-homogeneous samples. The mutual juxtaposition of phases in a mineral helps to trace the history of its formation. The details of spatial arrangement of particles in a multi-particle material are significant for its properties.

Modern techniques make it possible to collect diffraction data from local areas by scanning large particles, and photographs of these particles are a natural addition to the multiple diffraction patterns. Also, when integral 1D diffraction patterns are collected from a multi-phase sample by grinding and homogenizing it, having a photograph of the original large sample is very informative, supporting the conclusions of the phase analysis derived from Rietveld refinement. As an example, see Fig. 41 of Dinnebier & Friese (2003 ▸) presenting a microphotograph of a cement clinker sample containing five phases (alite, belite, ferrite, aluminate, periclase) and the corresponding 1D powder diffraction pattern used for a quantitative Rietveld refinement (also available at https://www.fkf.mpg.de/233448/xrd.pdf).

**Figure 18:**
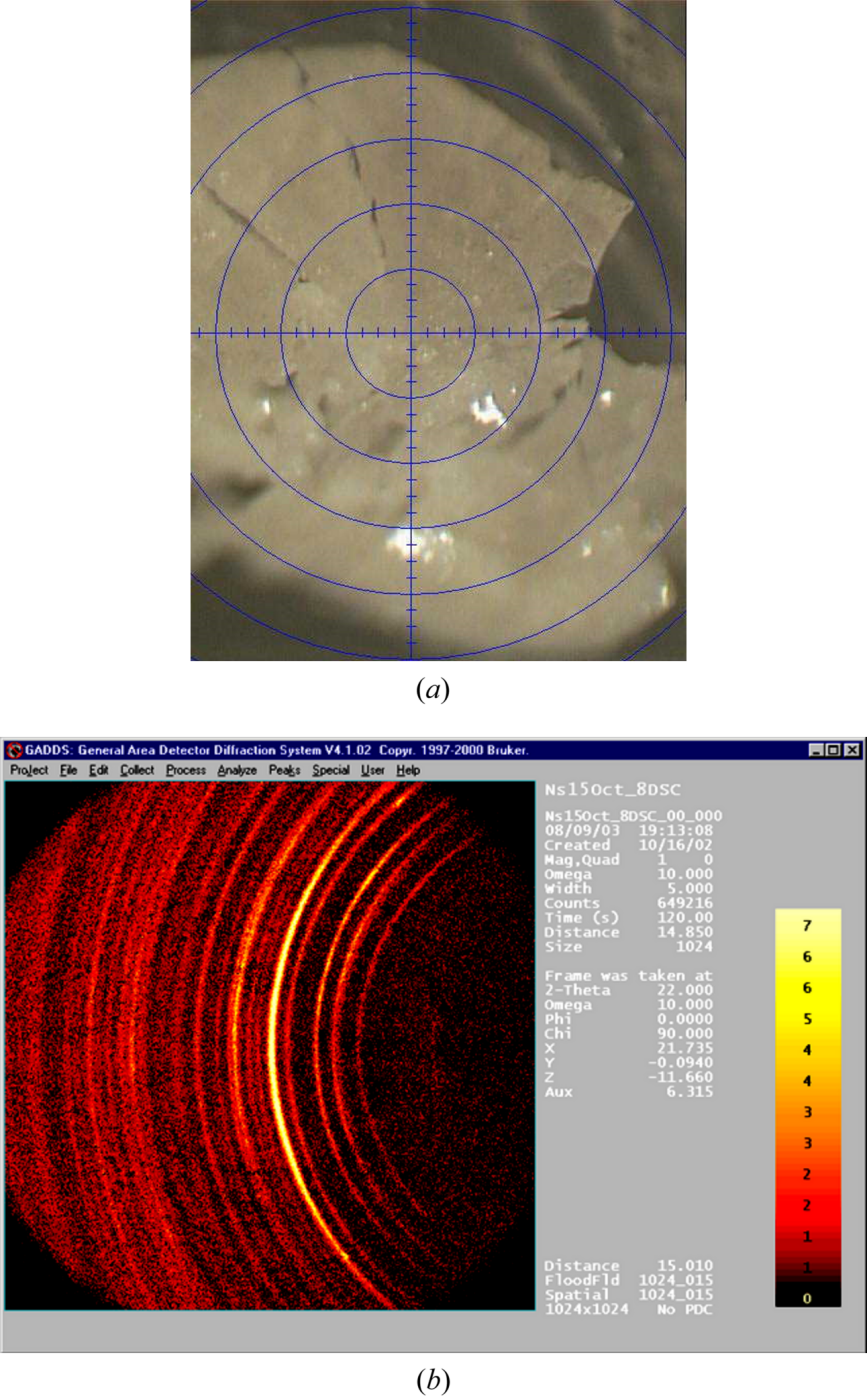
(*a*) A single crystal of sulfathiazole polymorph III after having transformed completely into polymorph I. The pseudomorph preserves the initial shape. There is no evidence of melting. The visible cracks appeared during the polymorphic transition. (*b*) A diffraction pattern of the pseudomorph shown in (*a*). [The plot is from the Boldyreva research group archive. Data used for this plot were discussed by Drebushchak *et al.* (2008 ▸).]

The products of many solid-state transformations (polymorphic transitions, dehydration, thermal decomposition) may look like a single crystal but be built of many crystallites. They therefore give diffraction patterns which are typical for fine powders (Fig. 18 ▸). A photograph of the ‘pseudomorph’ from which the powder diffraction pattern was collected and details of the orientation of the pseudomorph with respect to the incident X-ray beam and detector may contain valuable information on the order–disorder relationship of the transformation product with respect to the parent single crystal. This information may serve as a key for understanding the mechanism of the transformation needed to control the characteristics of the product.

**Figure 19:**
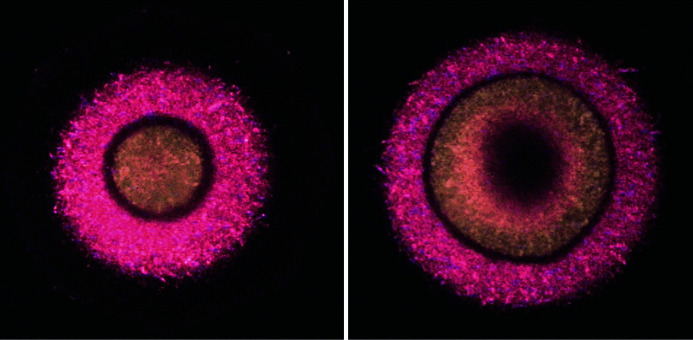
A sample of HgI_2_ uniaxially squeezed between DAC culets, with stress increased from the left to right photographs. The color changes in HgI_2_ mark phase III (to 0.4 GPa), phase IV (to 1.3 GPa), phase VI (to 7.6 GPa) and phase VII at the center of the right photograph. Reproduced with permission of the International Union of Crystallography from Katrusiak (2008 ▸); data from Hostettler & Schwarzenbach (2005 ▸).

High-pressure research is one of the fields where preserving the image of the sample may be of utmost importance. For samples studied *in situ* under non-ambient temperature and/or pressure conditions, an image of the sample can indicate the non-uniformity of temperature and/or pressure distribution in the sample chamber. Fig. 19 ▸ provides such an example.

**Figure 20:**
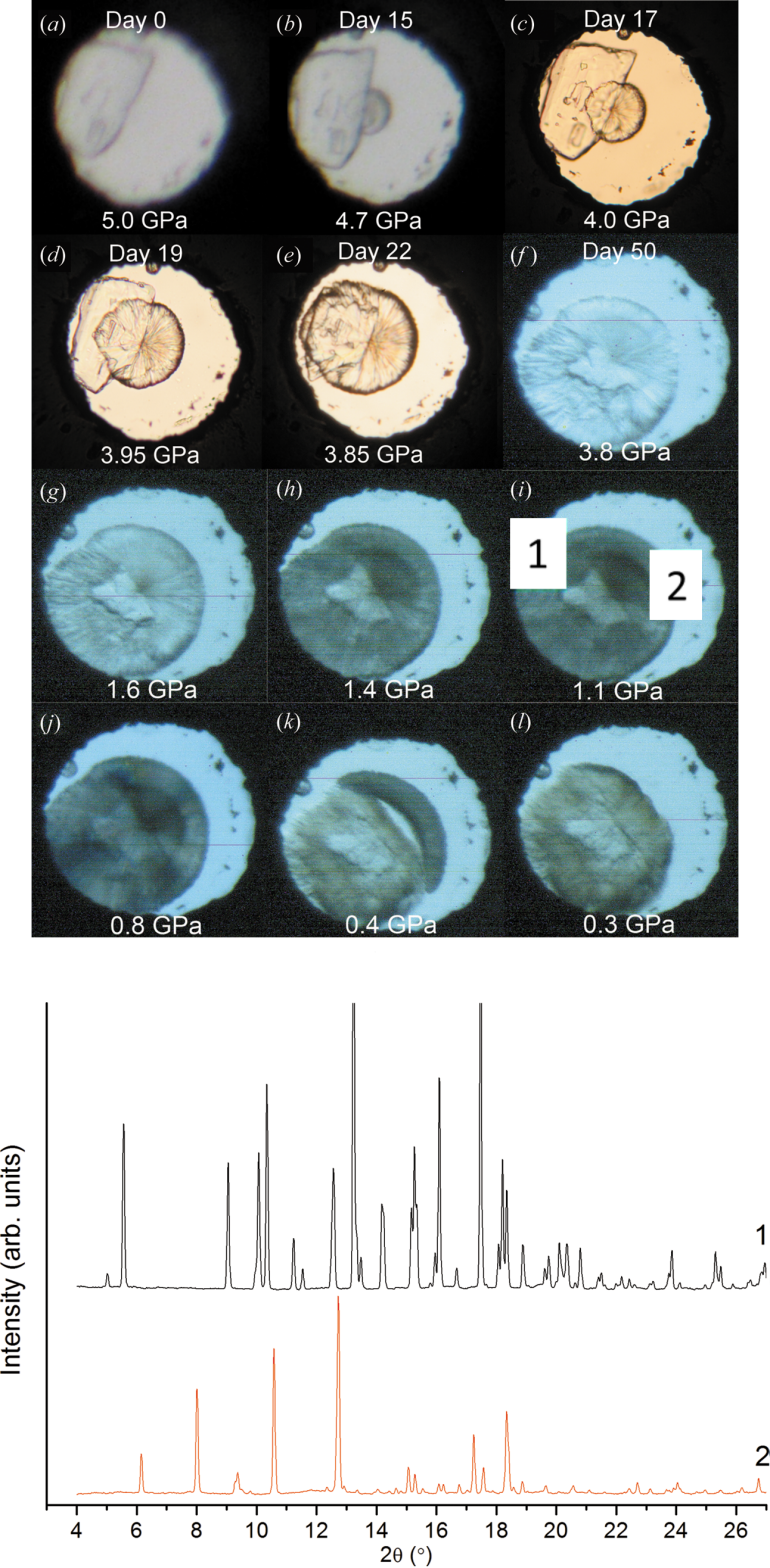
A series of photographs of a sample of l-alanine immersed in a methanol–ethanol–water mixture in a DAC, showing a sequence of transformations on decompression from 5 GPa to ambient pressure. Top: the sample as viewed in an optical microscope. First a single crystal of l-alanine, then polycrystalline phase 1 (a solvate) and phase 2 (a polymorph of l-alanine) formed on desolvation of phase 1 on decompression. Bottom: powder diffraction patterns collected from different parts of the sample in the left photographs (already converted from the 2D to 1D format with background eliminated). Reproduced with permission of the International Union of Crystallography from Tumanov *et al.* (2010 ▸).

Fig. 20 ▸ shows two powder diffraction patterns collected from different sites in a high-pressure cell that correspond to two co-existing high-pressure phases, marked 1 and 2. Phase 1 is a polycrystalline solvate that is formed if a single crystal of l-alanine immersed in a methanol–ethanol–water mixture is compressed to 6 GPa, then decompressed to 5 GPa and subsequently kept at this pressure for several weeks. Phase 2 is a polymorph of l-alanine II that is formed on desolvation of phase 1 on decompression to 0.4 GPa and then transforms to l-alanine I on complete decompression to ambient pressure (Tumanov *et al.*, 2010 ▸). The photographs illustrate clearly that the two high-pressure phases co-exist in certain pressure ranges with each other and with the ambient-pressure polymorph of l-alanine, and still the powder diffraction patterns, being collected locally, contain the diffraction maxima of only one phase each.

## 13. Why revisit old data?

Revisiting original old data is not very common after these data have already been successfully published and even reproduced by other research teams. Still, it can happen that some ‘minor’ details in the data remain unclear and either keep ‘disturbing’ the authors of the data or attract the attention of other groups, should they have access to these data. A detailed study of the polymorphism of l-serine at high pressures can be used as an illustration.

The same sequence of reversible cooperative transformations of ambient-pressure phase I to high-pressure phases II and then III was documented repeatedly and reproducibly by two research groups. The single crystal was preserved during the series of transformations, and therefore the structures of the high-pressure phases could be solved on the basis of either single-crystal or powder diffraction data and the results compared. The studies were carried out by single-crystal X-ray diffraction with a laboratory four-circle diffractometer with a point detector (Boldyreva *et al.*, 2006 ▸; Drebushchak *et al.*, 2006 ▸) and a laboratory X-ray diffractometer with an area detector (Moggach *et al.*, 2005 ▸), as well as by synchrotron X-ray powder diffraction using a 2D detector (Boldyreva *et al.*, 2006 ▸) and by neutron powder diffraction (Moggach *et al.*, 2006 ▸). All the studies gave the same structural models for the high-pressure phases, l-serine II and l-serine III.

**Figure 21:**
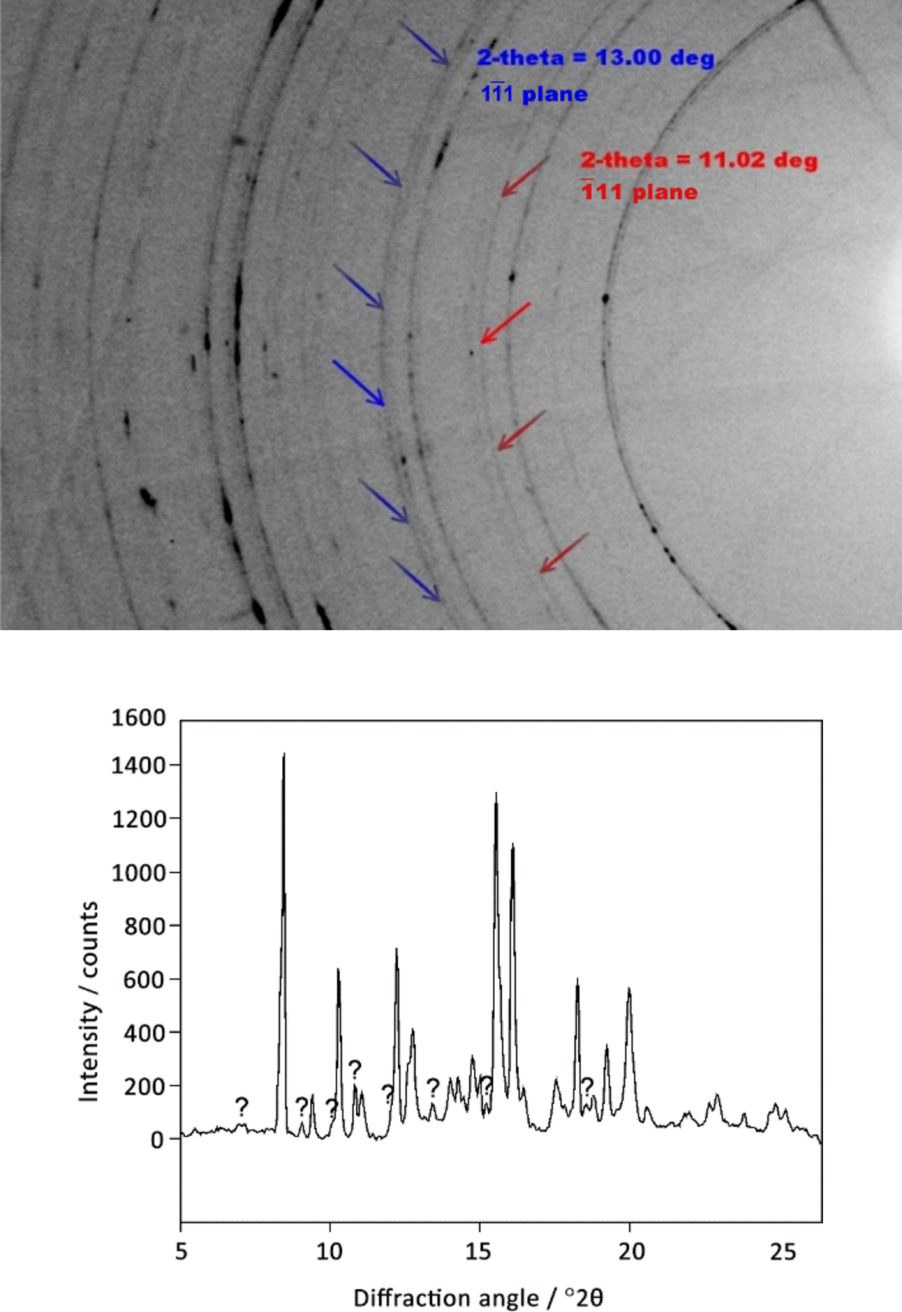
Top: a fragment of the 2D pattern collected from a powder sample of l-serine III in a DAC at a synchrotron experiment with some unidentified peaks. Bottom: the same pattern after being converted to a 1D format (the peaks that could not be assigned to l-serine III are marked ‘?’). Lower figure reproduced from Boldyreva *et al.* (2006 ▸) with permission from Elsevier.

There was, however, a problem that remained unsolved. Even though the match between the experimental data and the structural models was generally good, there were some weak diffraction peaks in the powder pattern of phase III that did not belong to any of the known phases of l-serine. A 2D pattern collected for the powder sample confirmed that these maxima corresponded to a powder. And this was obviously not an impurity introduced into the sample (the cell was closed) but a phase formed on compression and disappearing on decompression. Its structure could not be solved, and so question marks were left in the publication when describing the peaks that could not be assigned to any of the known phases (Fig. 21 ▸) (Boldyreva *et al.*, 2006 ▸).

**Figure 22:**
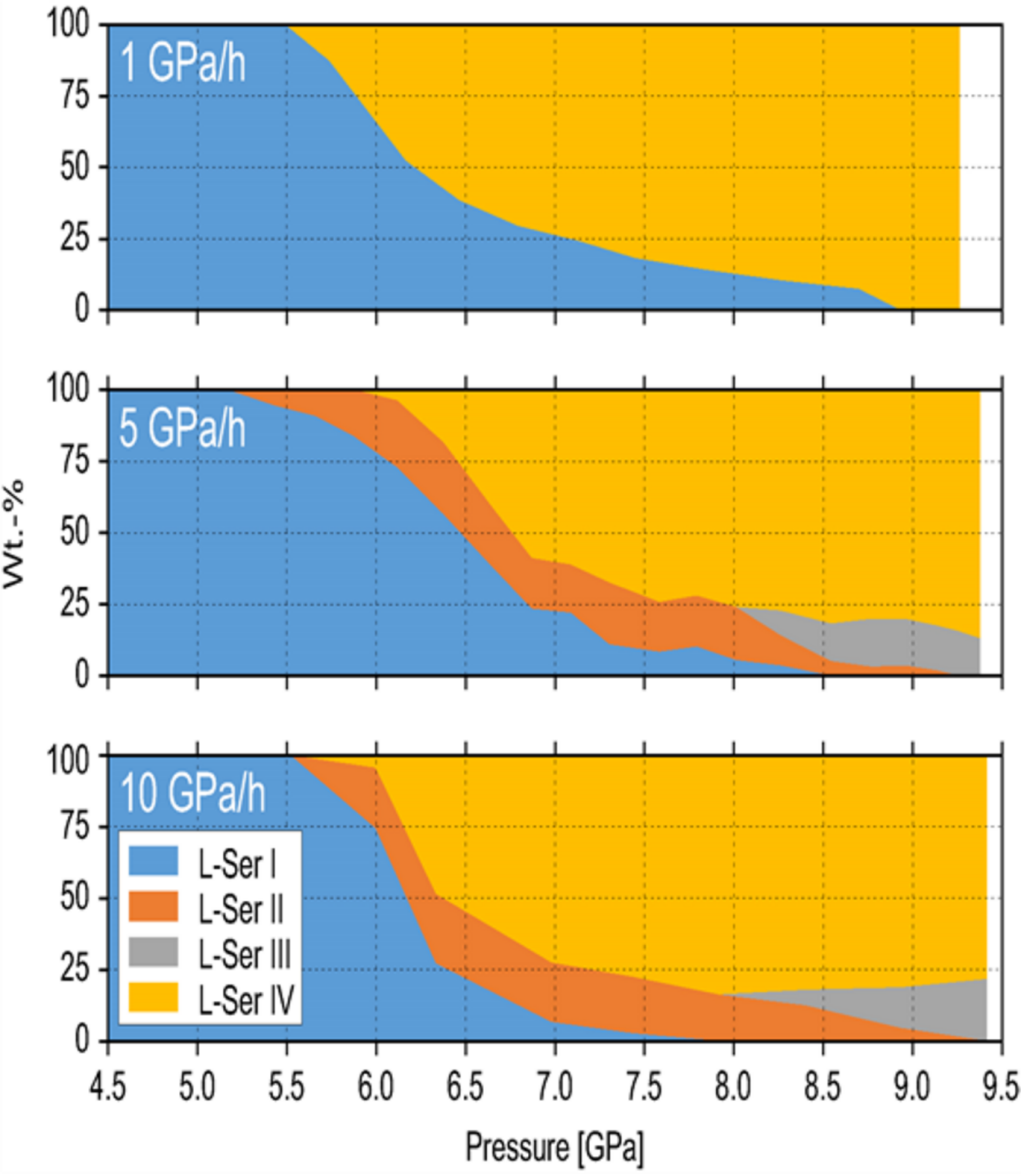
Relative contents of four polymorphs of l-serine at various pressures depending on the pressure-increase rate. Figure prepared using data published by Fisch *et al.* (2015*a* ▸).

Ten years later, another powder diffraction experiment with controlled pressure-increase rate made it possible to observe another, parallel, route for a high-pressure transformation: conversion of l-serine I directly to l-serine IV, avoiding phases II and III. The structure of l-serine IV could be solved and refined from these powder diffraction data. The relative amounts of phases I, II, III and IV could be controlled reproducibly by controlling the pressure-increase rate (Fig. 22 ▸) (Fisch *et al.*, 2015*a* ▸). With these new data available, the raw diffraction data collected ten years earlier at another synchrotron source, from another sample, in a different type of DAC and with another set-up for registering the diffraction pattern, could be revisited and reprocessed. This meant that all the previously unexplained ‘extra’ diffraction maxima in the pattern of ‘phase III’ could be identified as belonging to phase IV. So, the powder sample studied in 2005 and described as polymorph l-serine III turned out to be a two-phase system. Moreover, with new data available it was possible to process this old diffraction pattern in a new way and to find quantitatively the ratio of III and IV phases present in that old sample. Storing the raw data which were reusable and replicable, we could in fact add new knowledge on the system that seemed to be so well studied already. Using the data of the two studies was in fact synergetic. The 2D data published by Boldyreva *et al.* (2006 ▸) indicated clearly the presence of two polycrystalline high-pressure phases, but the crystal structure of the phase present as a minor component could not be solved. The set-up with a 1D detector used in the research by Fisch *et al.* (2015*a* ▸) could offer much better resolved peaks, providing up to ten times better angular resolution, which was of advantage for indexing, for lattice parameter refinement, and even for structure refinement and solution (Fisch *et al.*, 2015*b* ▸).

## 14. Conclusions

Although data are not information and knowledge is not understanding, no true knowledge and understanding are possible without access to reliable primary data. This access may be needed more than once, and often much later than data have been collected.

It remains disputable which ‘raw data’ should be shared and/or preserved. We are well aware of the fact that, for the sake of saving storage resources, many researchers advocate that fully processed powder diffraction data should be routinely deposited and shared rather than the data before the instrument corrections. They suggest that the firmware corrections can be fully trusted and it is after those firmware corrections that the raw diffraction images are recommended to be preserved (Aranda, 2018 ▸). Our own experience of studying complex samples – often obtained under non-ambient temperature and pressure conditions as metastable, defect, disordered, heterogeneous and multi-phase samples, which are not always easy to reproduce and may even be unique – has taught us the opposite: storing already processed data may be of little help for future research. Conversely, access to the primary data (complemented by all the necessary metadata needed to reprocess them once again at a new level) can be a source of new knowledge even years after the data have been collected originally.

The rising interest in machine-learning techniques brings a new dimension to the problem of making already collected powder diffraction data easily findable, accessible and reusable (Suzuki, 2022 ▸; Surdu & Győrgy, 2023 ▸). With the progress in modern instruments, data collection has become very fast and can be performed in automatic and remote modes. Data processing, however, takes orders of magnitude more time than data collection and still demands much human creative involvement. With the large number of powder patterns collected in academia, in industry, in laboratories and at large-scale facilities, it becomes very attractive to process data automatically (Arcelus *et al.*, 2024 ▸; Karnevskiy *et al.*, 2024 ▸). Attempts are being made to use machine-learning methods for phase identification (Schuetzke *et al.*, 2021 ▸), qualitative phase analysis (Greasley & Hosein, 2023 ▸), perturbation and artifact identification, masking (Lee *et al.*, 2023 ▸; Yanxon *et al.*, 2023 ▸), structure solution (Niitsu *et al.*, 2024 ▸), symmetry identification, property prediction, and low-dimensional embedding (Suzuki, 2022 ▸). Machine-learning (neural-network) techniques can determine the diffraction peaks buried in noise and allow deconvolution of overlapped peaks, resulting in crystal-structure determination by Rietveld analysis (Niitsu *et al.*, 2024 ▸). Despite the enormous number of diffraction patterns available worldwide, not all of them turn out to be suitable for training machine-learning models (Greasley & Hosein, 2023 ▸). In order to generate a reliable error-free automatic algorithm, one needs reliable and error-free samples for educating the machine. It is already clear that full-profile X-ray diffraction patterns are needed as descriptors, in order to minimize human intervention, that is, not to apply knowledge-based data selection or handcrafted feature engineering (Lee *et al.*, 2022 ▸). This is why so much attention should be paid to achieving the best possible data quality and completeness when collecting and depositing powder diffraction patterns. Some possible origins of problems are discussed throughout this manuscript. We have tried to summarize this discussion in Table 3 ▸. The main general problem is that many experimental details are either completely neglected when collecting data or not deposited with the data, so that it is not possible to revisit the same data later in order to reveal overlooked errors or get any added knowledge.

Many years ago Professor J. D. Dunitz noted: ‘Diffraction data are trying to tell us something, but we have no time to listen – another overdue paper is waiting to be written.’ This statement sounds today even more timely. There is an overwhelming amount of data being collected, and there is no deficit of papers reporting new results. What is missing in our opinion is attention to how data were obtained and processed. A critical detailed analysis of data quality and reliability is important, in order to evaluate to what extent these data are reliable and correct and their interpretation is non-ambiguous. Making original raw data and all the details of the procedures of their processing and analysis accessible to other researchers, and also at a later time, will enable these data to be revisited. It will allow researchers to check and possibly to improve (or even to correct) their analysis, and potentially to see some features that remained unnoticed earlier and become visible after new data have been obtained, new methods of data analysis developed or simply a new team looks at the data with ‘fresh eyes’.

## Figures and Tables

**Table 1:** Information that can be derived from reduced diffraction data

	Single-crystal diffraction pattern	Powder diffraction pattern
Positions of Bragg peaks	Unit-cell parameters, space symmetry group	Unit-cell parameters, space symmetry group
Relative intensities of Bragg peaks	Atom types, atomic coordinates, symmetry, thermal motion	Atom types, atomic coordinates, symmetry, thermal motion, but also preferred orientation and type of disorder; positions + relative intensities of Bragg peaks in many cases can ‘fingerprint’ a known crystalline phase
Shapes of Bragg peaks	Strain, particle shape	Strain, size distribution of coherently diffracting domains (crystallites), presence of defects and their types, in some cases fingerprinting of a phase (when not possible without the analysis of peak shapes)
Additional peaks between Bragg peaks	Twins, modulation, foreign phases in the sample or in the material of a sample environment (*e.g.* for data collection from a crystal in a pressure cell)	Foreign phases in the sample or in the material of a sample environment (*e.g.* for data collection from a crystal in a pressure cell), modulation
Diffuse scattering between Bragg peaks	Disorder, lattice dynamics	Disorder, lattice dynamics
Background	Fluorescence, contribution from the sample chamber (*e.g.* in high-pressure crystallography or for a crystal in capillary)	Disorder, lattice dynamics, fluorescence, content of amorphous phases

**Table 2:** Different levels of data

Primary data	Processed data	Reduced data
Raw detector data (pixel, intensity, detector characteristics)	2D image after corrections	Peak shapes, intensities, structure factors, unit-cell parameters, atomic coordinates, size of coherent scattering domains *etc.* (see Table 1 ▸) after integration and analysis
Calibration files for detector/measurement	1D pattern after corrections and radial integration	
Sample origin and history	Data after further corrections from non-sample contributions	
Sample environment (pellet, capillary, sample in a furnace, DAC, multi-anvil apparatus)		
Conditions (pressure, temperature, compression rate in the case of dynamic compression experiments, charging/discharging of a battery during *operando* studies *etc*.)		

**Table 3:** Some problems that may arise in looking at powder diffraction data when specific metadata are missing

		Affecting	
Missing metadata	Possible problem	Diffraction pattern	Information on the sample/the process
*Data collection*			
Source stability	Not constant/not continuous during data collection	Peak intensities	Potential problems with structural models and/or kinetic data
Radiation exposure; beam intensity and total time in the beam	Radiation damage difficult to estimate	Whole pattern, peak positions, intensities and shapes	Wrong conclusions on the structural parameters, phase composition, disorder, solid-state transformations
Spinning/rocking – applied or not, which parameters if applied	Not enough particle statistics/overestimated homogeneity of the diffraction rings	Relative peak intensities, peak shapes, underestimated inhomogeneity of the diffraction rings	Potential errors in structural model, size of coherently diffracting domains, strain and sample homogeneity; data collected without rocking for an equation of state, refined with a standard Lebail fitting, may be unsuitable for Rietveld refinement
	Periodic movement	Periodic background peaks changing in pattern	Wrong conclusions on the dynamics of the process
IRF calibrant	Unknown IRF	Peak shapes	Potential errors in size of coherently diffracting domains, strain
Beam shaper (slits, pinhole)	Unknown IRF, beam position	Peak positions, shapes	Potential errors in size of coherently diffracting domains, strain, unit-cell parameters
Sample holder	Different size, scattering	Peak positions, shapes, background	Potential errors in size of coherently diffracting domains, strain, unit-cell parameters
Sample chamber parameters	Possible problems with absorption and shadowing corrections	Wrong peak intensities, background	Serious problems with analyzing data – from errors in a structural model to the complete inability to suggest a model
For multi-temperature/multi-pressure measurement – protocol of changing temperature/pressure	Radiation damage problems may be overlooked; kinetic control of the processes may be not revealed	Changes in the diffraction patterns (position of peaks, their intensities and shapes) can depend on these protocols	Equations of state, wrong values of thermal expansion and isothermal compressibilities; different processes giving different products may be observed

*Data processing*			
Applied angular calibration	Offset	Wrong peak positions	Errors in unit-cell parameters
Applied flatfield	Fluorescence	Increased background, bias in data, non-statistical noise	Errors in structural model: wrong error estimates, at times wrong intensities and possibly wrong refined parameters
Details of masking	Scattering from other sources, defect pixels	Background, peak intensities, extra peaks	Errors in structural model may be difficult or impossible to reveal; important information on presence of phases may be overlooked
Details of absorption correction	Absorption by sample itself, sample chamber materials or sample environment	Background, peak intensities	Errors in structural model may be difficult or impossible to reveal
Details of 2D →1D conversion	Errors may be difficult or impossible to reveal		
Details of quantitative phase analysis procedure	Errors may be difficult or impossible to reveal		
Details of analysis of size of coherently scattering domains and strain	Errors may be difficult or impossible to reveal		
Details of structure solution and refinement procedure	Errors in structural model may be difficult or impossible to reveal		

*Data deposition*			
Sample images with information on from which sample site each pattern was collected	Impossible to relate images with diffraction patterns; potential problems with recognizing phases; information about sample/process may be incomplete; potential problems with comparing the results of several different experiments		
Complete pattern (peak profiles for 1D pattern; 2D diffraction pattern if a 2D detector was used; all the patterns for multiple data collections)	Impossible to solve and refine crystal structure, to analyze the size of coherently scattering domains, to analyze strain, to study defects and disordering, to reveal amorphous states; information on sample texture or preferred orientation lost; identification of known phases (fingerprinting) may be impossible; potential problems with comparing the results of several different experiments		
Raw data before masking and introducing any corrections	Impossible to work with uncorrected/unmasked data independently, in order to improve the results or get new knowledge		
Software used to process raw data introducing corrections and masking	Impossible to check the quality of corrections and masking independently; impossible to work with uncorrected/unmasked data independently		

## Footnotes

1 Different researchers understand differently which data should be considered as ‘raw’. See more on this in Section 3.
